# Hydrogels for Oral Tissue Engineering: Challenges and Opportunities

**DOI:** 10.3390/molecules28093946

**Published:** 2023-05-07

**Authors:** Anfu Chen, Shuhua Deng, Jindi Lai, Jing Li, Weijia Chen, Swastina Nath Varma, Jingjing Zhang, Caihong Lei, Chaozong Liu, Lijia Huang

**Affiliations:** 1 Guangdong Provincial Key Laboratory of Functional Soft Condensed Matter, School of Materials and Energy, Guangdong University of Technology, Guangzhou 510006, China; anfuchen@gdut.edu.cn (A.C.);; 2 Guangdong Provincial Key Laboratory of Stomatology, Department of Operative Dentistry and Endodontics, Guanghua School of Stomatology, Hospital of Stomatology, Sun Yat-Sen University, Guangzhou 510275, China; 3 Institute of Orthopaedics and Musculoskeletal Science, Division of Surgery and Interventional Science, University College London, Royal National Orthopaedic Hospital, London HA4 4LP, UK

**Keywords:** tissue engineering, hydrogels, dental pulp, periodontium, mandible

## Abstract

Oral health is crucial to daily life, yet many people worldwide suffer from oral diseases. With the development of oral tissue engineering, there is a growing demand for dental biomaterials. Addressing oral diseases often requires a two-fold approach: fighting bacterial infections and promoting tissue growth. Hydrogels are promising tissue engineering biomaterials that show great potential for oral tissue regeneration and drug delivery. In this review, we present a classification of hydrogels commonly used in dental research, including natural and synthetic hydrogels. Furthermore, recent applications of these hydrogels in endodontic restorations, periodontal tissues, mandibular and oral soft tissue restorations, and related clinical studies are also discussed, including various antimicrobial and tissue growth promotion strategies used in the dental applications of hydrogels. While hydrogels have been increasingly studied in oral tissue engineering, there are still some challenges that need to be addressed for satisfactory clinical outcomes. This paper summarizes the current issues in the abovementioned application areas and discusses possible future developments.

## 1. Introduction

The oral cavity is an integral component of the digestive system, containing several significant anatomical components made up of various soft and hard tissues, such as teeth, oral mucosa, periodontal tissues, maxilla, and mandible. Additionally, the oral cavity is simultaneously populated largely by over 700 kinds of microorganisms, creating a complex ecological niche that directly impacts oral health [1]. Most researchers agree that dysbiosis of the microflora, rather than a particular species of bacteria, is the root cause of oral infectious disorders, including dental caries, periodontitis, peri-implantitis, and oral candidiasis [2]. Maintaining good oral health is critical in fending off periodontal diseases and dental caries, which can lead to more serious health problems, such as endocarditis, diabetes mellitus, and Alzheimer’s disease. Therefore, the prevention and treatment of these oral illnesses have thus received significant attention [3]. Oral diseases are pathological changes that occur in the soft and hard structures of the oral cavity and maxillofacial region [4]. The most common oral diseases include dental caries, periodontitis, pulp necrosis, oral mucositis, and jaw abnormalities [5]. It is estimated that more than 3~5 billion people worldwide experience chronic oral disorders that progress over time, starting in early infancy and continuing throughout adolescence, adulthood, and later life [6]. In many countries, oral disorders place a significant health burden on individuals, causing various degrees of pain, discomfort, disfigurement, and even death in some cases. Given the complex and diverse nature of oral health issues, there seems to be an endless need for dental biomaterials that can effectively interact with a range of tissues, from soft gum tissue to hard bone tissue. Moreover, dental biomaterials must be able to withstand the challenges posed by the oral environment, including abrupt temperature changes, pH fluctuations caused by saliva and biofilms, and the presence of various types of bacteria [7]. Consequently, there has been increasing research on developing effective treatments for oral illnesses. Bacterial infections frequently lead to oral disorders [8], and there are two main approaches for treating them. One aims to combat the infections, while the other aims to promote tissue regeneration. For repairing jawbone defects, autologous bone grafting is the most common treatment method currently used. However, autogenous bone is a limited source and cannot be reshaped to fit the defect. For periodontitis and oral mucositis, the prevention of bacterial infection is critical. Systemic administration has long been the primary treatment for the infectious diseases of the oral cavity. However, systemic administration may cause issues such as drug resistance and liver toxicity. For pulpal necrosis, regenerative root canal therapy is the mainstream treatment method, and the preferred scaffold for this therapy is an injectable biomaterial. However, there is no consensus on the most suitable injectable scaffold for endodontic treatment. Hydrogel, as a biocompatible material that can easily change shape, performs excellently in both drug delivery and tissue regeneration. Hydrogels usually possess a porous structure due to their internal network that forms many tiny pores and voids. The size and shape of these pores and voids can be controlled by regulating the conditions and preparation process during gel synthesis. By regulating the structure of the pores, it is possible to match them with surrounding tissues, promoting cell adhesion and growth [9,10]. Moreover, hydrogels exhibit excellent antimicrobial properties in oral clinical applications. Firstly, they physically isolate bacteria, preventing the invasion and spread of harmful microbes. Secondly, hydrogels act as effective drug release carriers, facilitating the delivery of various antimicrobial substances to achieve their desired effects. Recently, there has been considerable research on stimuli-responsive hydrogels. These hydrogels can respond to external physical, chemical, and biological stimuli, triggering the release of antimicrobial agents. Additionally, these hydrogels can exhibit antimicrobial properties through responsive physical and chemical properties, such as by modulating pore structure and regulating humidity. Therefore, hydrogel has become a promising material for the treatment of oral diseases.

Hydrogels have a wide range of applications in dental science research, from treating oral diseases to reconstructing tissues. They are amphiphilic polymer networks that can be created in situ through physical or chemical cross-linking, and have a high fluid absorption capacity without compromising their structural integrity [11,12]. Hydrogels are considered potential biomaterials because they mimic the biochemistry of the extracellular matrix (ECM) [13], and can be used for the transport of drugs and cells [14]. Synthetic polymer hydrogels with clearly defined chemical structures, molecular weights, superior mechanical strength, and customizable microstructures can be prepared using well-monitored synthetic processes [15]. However, synthetic polymer hydrogels are typically non-biocompatible and non-biodegradable, making them unsuitable for supporting cells [16]. Natural polymer hydrogels derived from plants, animals, or microorganisms have recently attracted a lot of interest as ideal dental biomaterials [17]. Natural hydrogels have good biocompatibility and biodegradability, but their poor mechanical properties, uncontrollable degradation rates, and potential for immune reactions limit their practical applications. Therefore, synthetic–natural composite hydrogels have been proposed to meet the requirement for potential dental applications. Composite hydrogels are the most popular injectable matrices for cellular delivery [18]. Hydrogels can create three-dimensional (3D) matrices for the encapsulation of sensitive bioactive compounds or living cells. The combination of various natural and synthetic hydrogels holds promise for achieving the regeneration and repair of craniofacial and oral tissues. More importantly, hydrogels are stimulus-responsive, meaning they can change their structure, physical properties, chemical composition, and elasticity modulus in response to external stimuli such as changes in temperature, pH, ionic strength, light, magnetic, electrical, or mechanical signals [19]. Natural hydrogels such as alginate, hyaluronic acid (HA), gelatin, collagen, and chitosan (CS) are often used, while synthetic hydrogels such as polylactic acid (PLA), polyethylene glycol (PEG), and gelatin methacryloyl (GelMA) are frequently used in biomedical applications [20].

**Figure 1:**
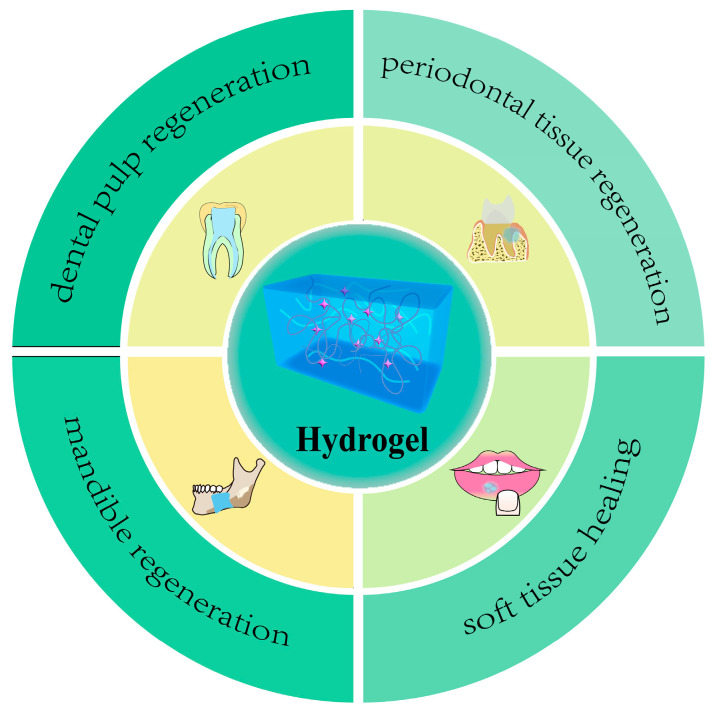
Applications of hydrogel in oral cavity.

There has been a significant amount of research on the use of hydrogels for treating oral disorders. The objective of this review is to examine the different forms of hydrogels that have recently become popular in dentistry and their uses in oral tissue engineering. We will discuss the latest advancements in pulp regeneration, periodontal tissue regeneration, mandibular bone repair, and oral soft tissue healing using five types of natural hydrogels and three types of synthetic hydrogels. Figure 1 illustrates these applications. Pulpal and periapical diseases are among the most prevalent inflammatory oral diseases, with reported worldwide prevalence ranging from 16% to 86% [21,22,23]. Epidemiological evidence has demonstrated that approximately 20% to 50% of the global population is affected by periodontal-associated diseases, and almost 10% of the global population suffers from severe periodontitis [24]. Oral lichen planus (OLP) and recurrent aphthous stomatitis (RAS, also known as oral ulcers) are diseases that mainly affect the soft tissues of the oral cavity [25]. They affect up to 25% of young adults and an even higher percentage of children [26]. It is clear that diseases related to dental pulp, periodontal tissue, and oral soft tissue are very common. Moreover, mandibular defects caused by various reasons are currently difficult problems to solve. Therefore, this review will discuss the progress of research on hydrogels in the four areas mentioned above. The injectability of hydrogel scaffolds has several benefits in creating endodontic tissue, which result from the small size and intricate structure of the root canal. The injected hydrogels can reach all the root canals [27]. As a result, investigations on the use of hydrogel materials in endodontic tissue engineering are more prevalent among biomaterials. Hydrogels have garnered interest in the fields of periodontal tissue engineering and maxillofacial bone tissue engineering due to their plasticity and efficient absorption into surrounding tissues. In addition, hydrogels can conform to the oral mucosa and exhibit an appropriate elasticity modulus similar to that of soft tissues. As a result, recent studies have focused on the development of hydrogel oral mucosal patches.

## 2. Classification of Hydrogels

### 2.1. Natural Hydrogels

Hydrogels are polymer networks with a high water absorption capacity, formed by physical or chemical cross-linking. They can be categorized into two groups based on the source of their raw materials: natural hydrogels and synthetic hydrogels. Natural hydrogels include the collagen and gelatin extracted from animal proteins [28], the HA commonly found in animal epithelium and connective tissue [29], and alginate derived from the cytoplasm and cell wall of algae and seaweeds [30]. These biomaterials are non-toxic, highly biosafe, and biocompatible, making them suitable for various biomedical applications [31,32,33]. In the following section, we describe several hydrogels commonly used in the biomedical field.

#### 2.1.1. Collagen-Based Hydrogels

Collagen, the main component of the ECM in many mammalian tissues, provides support and protection to the organism and its organs due to its excellent coagulation effect. Collagen-based hydrogels have attracted significant attention from researchers, owing to their weak immunogenicity and good biocompatibility. These hydrogels have been widely applied in cartilage repair, dentistry, drug delivery, and corneal transplantation, with rapid development in recent years [34,35,36,37]. Xie et al. prepared oxidized starch cross-linked porous collagen hydrogels with good mechanical properties and investigated their influence on biological regulation. The results showed that these hydrogels have great potential as biological scaffold materials in medicine [38]. Fahimeh et al. further demonstrated that collagen-based hydrogels can promote the growth and differentiation of oral fibroblasts and epithelial cells [39].

#### 2.1.2. Hyaluronic Acid (HA) Hydrogels

HA, a nonsulfated glycosaminoglycan found in all connective tissue ECM, is one of the most commonly used natural polymers today [40,41]. Studies suggest that HA plays a crucial role in biological processes such as angiogenesis, ECM structure, inflammation, and wound healing. Moreover, HA derivatives have been successfully employed as scaffold biomaterials for chondrocyte development, bone regeneration, and skin tissue regeneration [42,43]. Singh et al. injected HA hydrogel into root canals as a scaffold for the regenerative root canal treatment of necrotic teeth and achieved successful induction of continued root development [44].

#### 2.1.3. Gelatin Hydrogels

Gelatin is a hydrophilic polymer that possesses excellent sol–gel transition properties and biocompatibility, making it a versatile material in the field of hydrogels [45]. Hydrogels made from gelatin as a matrix can mimic various tissue characteristics and allow for the tailoring of hydrogel properties, such as mechanics and degradation, to suit a wide range of biomedical applications [46,47]. Xie et al. prepared a self-shrinking wound dressing using the oxidized starch of shape memory hydrogels, which showed through ex vivo experiments that shape memory was activated at conditions similar to human physiological temperature, thus achieving atraumatic mouth closure [48]. Customized hydrogels that mimic natural cells have been found to enhance endodontic treatment, while gelatin methacrylate-based hydrogels with modifiable physical and mechanical properties have been identified as an effective strategy to promote endodontic regeneration [49,50]. Researchers have also demonstrated that the dental light-cured preparation of GelMA can maintain the viability of adult dentin cells, thereby promoting its application in dentistry [51]. Furthermore, Han et al. used methacrylic anhydride-modified gelatin to obtain photocrosslinkable GelMA, which exhibited excellent mechanical properties and thermal stability. In vitro cell culture experiments revealed that the hydrogels also exhibited remarkable bioactivity by maintaining the chondrocyte phenotype and promoting cell adhesion and proliferation [52].

#### 2.1.4. Alginate Hydrogels

Alginate, a natural polysaccharide isolated from brown algae or bacteria, finds wide application in tissue engineering [53,54]. Its intrinsic structure is similar to natural ECM, making it an excellent choice for biocompatible scaffolds. Pan et al. successfully regenerated alveolar skeletal and soft tissues using a bionic polysaccharide hydrogel/hydroxyapatite composite scaffold, thus presenting a new approach for clinical bone defect repair [55]. The scaffold material prepared from alginate showed excellent dental differentiation ability, while the alginate and hydroxyapatite composites can induce differentiation of periodontal stem cells in vitro [56]. To promote the attachment and proliferation of endothelial cells and to induce the expression of angiogenic genes in endothelial cells, Xiong et al. developed negatively charged alginate gels for cell delivery [57].

#### 2.1.5. Chitosan (CS) Hydrogels

CS, a long-chain cationic polysaccharide, is a deacetylated derivative of chitin [58]. Its wide range of biological properties, including antibacterial, anti-inflammatory, anticancer, and tissue repair properties, have been amply demonstrated. Additionally, CS has excellent drug-loading capacity in the form of nanoparticles and hydrogels. The reactive groups (e.g., –OH, –NH_2_) on the chitosan backbone allow for the production of multiple derivatives with the same properties as the parent polymer, but with enhanced biocompatibility and non-toxicity [59,60,61]. These advantageous properties make CS a valuable material for use in complex oral situations.

### 2.2. Synthetic Hydrogels

Synthetic hydrogels are created through chemical reactions, as the name implies. Compared to natural hydrogels with poor mechanical properties, stability, and low bactericidal efficiency, synthetic polymer hydrogels have high molecular weight and stable mechanical properties. Moreover, the structural morphology of synthetic hydrogels can be tailored according to their intended functionality and degradability. Therefore, they can be designed with advantages such as low degradability, efficient gel formation, and a long service life. However, synthetic polymer hydrogels are typically not antimicrobial and require antimicrobial treatment [62]. Currently, synthetic hydrogels are usually made from synthetic polymers derived from natural resources, such as PLA, PEG, and GelMA [63].

#### 2.2.1. Polylactic Acid (PLA)

PLA, a biodegradable synthetic polymer derived from abundant resources, is widely used in biomedical applications due to its excellent biocompatibility, low toxicity, biodegradability, ease of processing, and environmental friendliness [64,65]. PLA can be blended with other natural and synthetic polymers to create biodegradable PLA hydrogels [63]. Moreover, PLA can be copolymerized with synthetic polymers such as PEG to form PLA–PEG hydrogels, which have been shown to promote the differentiation of dental pulp stem cells in vitro [66]. Organic polymers derived from nature, such as polysaccharides and peptides, have also been incorporated into PLA and created hydrogels using physical or chemical techniques for use in tissue engineering, drug delivery, and wound healing [63]. For example, Sood et al. created carboxymethylcellulose-grafted poly(lactic acid-co-glycolic acid) hydrogels with antibacterial properties for targeted medication delivery and antibiotics [67]. Additionally, PLA can be used as an antibiotic carrier to reduce bacteria in healing tooth tissues without affecting the viability of pulp stem cells [68].

#### 2.2.2. Polyethylene Glycol (PEG)

PEG is a widely used synthetic polymer in biology due to its non-immunogenic, non-toxic, biodegradable, and highly hydrophilic nature. PEG-based hydrogels offer unique advantages in drug delivery and controlled release [69]. However, they do not provide optimal conditions for cell survival, adhesion, and development because they are inherently biologically inert. To address this limitation, Chow et al. created a biodegradable hydrogel with bioactivity and good mechanical characteristics for tissue engineering using polyethylene glycol acrylate [70]. Similarly, Ma et al. developed an injectable cross-linked hydrogel made from GelMA/poly(ethylene glycol) dimethacrylate as a bioscaffold [71]. This scaffold provided a suitable environment for periodontal stem cells to grow and facilitated dental regeneration.

#### 2.2.3. Gelatin Methacryloyl (GelMA)

**Figure 2:**
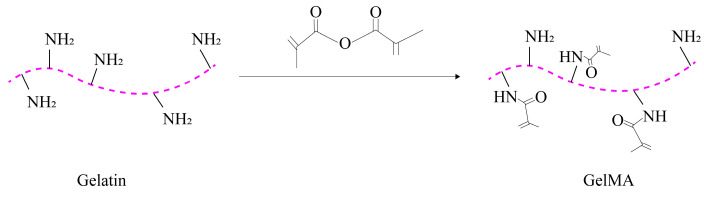
Synthesis process of GelMA.

GelMA is a photocrosslinked hydrogel that combines the properties of both natural and synthetic biomaterials. It is produced by adding methacrylate groups to gelatin [72]. The synthesis process of GelMA is illustrated in Figure 2.

The photocrosslinking ability of GelMA makes it a practical choice for gel production, cell encapsulation, and mechanical property modulation [73]. GelMA’s ability to precisely modify the 3D environment of a cell in tissue-engineered structures enables more predictable regeneration outcomes. The physical properties of GelMA hydrogels can be adjusted by altering the concentration of GelMA or photocrosslinker, which makes them suitable for encapsulating cells at 37 °C. Therefore, the GelMA has potential applications in dentistry, especially for encapsulating various types of stem cells.

## 3. Application of Hydrogels in Oral Tissue Engineering

### 3.1. Hydrogels for Dental Pulp Regeneration

Dental caries, trauma, and developmental malformations can lead to the irreversible destruction of the dental pulp, which plays a crucial role in maintaining immune defense, the sensory system, and regenerating the pulp–dentin complex [74,75,76]. The dental pulp is located inside the pulp cavity of the tooth and is protected by the non-resorbing dentin hard tissue. Following conventional root canal treatment for pulpal and periapical diseases, pulpless teeth lose their structural integrity, biological defense, and sensory capacity, which can result in the susceptibility of root fractures and poor long-term outcomes [77,78]. Thus, regenerating the dentin–pulp complex is of significant importance to restore the vitality of teeth, recover the biological function of teeth, and prolong the lifecycle of pulpless teeth.

Pulp regeneration, also known as regenerative endodontic treatment, was first proposed by Murray et al. in 2007. This ideal form of regenerative therapy aims to remove the diseased or necrotic pulp tissue and replace it with healthy, vital pulp tissue [79]. In 2016, the American Association of Endodontists formally defined pulp regeneration as the use of biological procedures to replace damaged tooth structures, including the pulp–dentin complex, root, and other structures, in order to form physiological and functional pulp-like tissue and restore biological function [80]. In general, pulp regeneration involves three interdependent elements: stem cells, biomaterials scaffolds, and bioactive molecules [81]. Through the comprehensive adjustment of these components, a repair microenvironment can be established to ensure the biological success of pulp regeneration [82].

**Figure 3:**
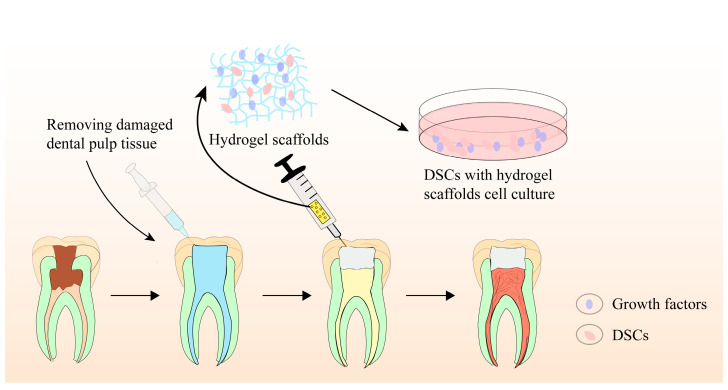
Schematic illustration of precise pulp regeneration procedure.

Biomaterial scaffolds play a crucial role in pulp regeneration by providing a 3D scaffold for stem cell adhesion, migration, proliferation, differentiation, and function. They not only regulate stem cell behaviors, intercellular and extracellular signaling, but also modulate the microenvironment, facilitating pulp–dentin complex regeneration. Recently, hydrogel-based scaffolds have been evaluated for tissue engineering-based pulp regeneration. These scaffolds have prominent biocompatibility, biodegradability, flexibility, elasticity, and ergonomic mechanical profiles, making them ideal candidates as cell or bioactive ingredient(s) delivery systems for promoting pulp–dentin complex regeneration. Figure 3 provides a schematic illustration of the precise pulp regeneration procedure.

Hydrogels can be classified into two categories based on the source of polymeric chain: natural and synthetic hydrogels. Natural hydrogels mimic natural peptides and possess favorable biocompatibility, but their mechanical characteristics are poor. In contrast, synthetic hydrogels exhibit distinct mechanical features and tunable physiochemical properties, but they are deficient in biocompatibility and biodegradability capacity [83].

Pankajakshan et al. evaluated the intrinsic ability of collagen hydrogels to modulate the endothelial and odontogenic differentiation of dental pulp stem cells (DPSCs) in regenerative endodontics [84]. The data strongly supported that collagen hydrogels with tunable stiffness promoted long-term cell survival and encouraged cell differentiation into specific lineages. Specifically, DPSCs cultured in collagen hydrogels with a stiffness of 235 Pa favored the expression of endothelial markers, while cells cultured in collagen matrices with a stiffness of 800 Pa demonstrated higher mineralization, with increased alkaline phosphatase (ALP) activity and Alizarin staining. In a study by Souron et al., pulp cells labeled with indium-111-oxine were loaded onto a collagen hydrogel scaffold and implanted into the pulp chamber space, and then tracked for at least three weeks. Histological analysis showed that active fibroblasts, new blood vessels, and nervous fibers were re-created in the pulp equivalents [85].

HA is a nonsulfated glycosaminoglycan component of the ECM of soft connective tissues [86]. As an outstanding candidate scaffold for pulp regeneration, HA has excellent biocompatibility, biodegradability, non-immunogenicity, and high water content [87]. Yang et al. proposed that HA hydrogels crosslinked with 1,4-butanediol diglycidyl ether may be a promising injectable scaffold for regenerating cartilage and dentin–pulp complex in a preliminary subcutaneous microenvironment [88]. Silva et al. investigated HA hydrogels incorporated with cellulose nanocrystals (CNCs) and reinforced with platelet lysate. The incorporation of CNCs significantly enhanced the stability and mechanical properties of HA hydrogels. It was found that resistance against hydrolytic and enzymatic degradation, the ability to recruit cells, and proangiogenic activity were significantly enhanced [89]. These results showed that the HA hydrogel exhibited great potential in the regeneration treatment of devitalized tissues.

The CS hydrogel is a promising biomaterial scaffold for regenerative endodontic treatment due to its bioactivity, biocompatibility, and capacity to blend with other bioactive ingredients. Bioactivity refers to properties of materials that trigger specific biological and chemical reactions, mainly at the interface between materials and biological tissues. Chitosan, for example, has the ability to promote cell adhesion, growth, proliferation, and differentiation. Additionally, chitosan possesses antibacterial activity. Several researchers have noted that CS-based hydrogels exhibit no biological toxicity toward various cell types and promote the proliferation and differentiation of stem cells, thereby facilitating the regeneration of pulp–dentin-like tissue [90,91]. However, other studies have proposed a different perspective, suggesting that CS-based scaffolds do not effectively encourage the regeneration of new mineralized tissues in devitalized endodontic space [92,93]. CS, as a second-generation polymer, exhibits the controlled biodegradation of the polymer chains [94]. Ongoing research is aimed at the definitive role of CS hydrogels in regenerative endodontics.

A clinical study was conducted to evaluate dentin bridges formed under overlay materials, using novel injectable processed dentin matrix hydrogels (TDMH), bio-dentin, and MTA, as assessed by CBCT imaging. The TDMH is a sodium alginate-based hydrogel, while both bio-dentin and MTA are the latest calcium silicate bioactive cements. Forty-five patients who had suffered accidental exposure to traumatic pulp were enrolled in the study. The results showed that TDMH was more effective in inducing the formation of dentin bridges than bio-dentin and MTA [95].

Natural hydrogels are known for their ability to mimic natural ingredients and exhibit super biocompatibility, but they carry the risk of immune reactions and poor mechanical properties. Synthetic hydrogels, on the other hand, can be engineered to possess adjustable mechanical profiles and microstructures. When equipped with bioactive molecules and cell-binding peptides, synthetic hydrogels are optimal candidates for tissue engineering applications. For example, Kuang et al. fabricated biocompatible and biodegradable PLA-based scaffolds and tested their regulatory role in dentin–pulp complex regeneration. The PLA-based scaffolds significantly promoted the proliferation and odontogenic differentiation of human dental pulp stem cells (hDPSCs) by enhancing the expression of ALP, osteocalcin, bone sialoprotein, collagen 1, and dentin sialophosphoprotein genes in in vitro experiment. Histological analysis demonstrated superior dentin-like tissue formation in vivo [96]. GelMA is another widely used synthetic hydrogel for endodontic regeneration, especially when modified with bioactive molecules. When GelMA hydrogels are encapsulated with hDPSCs and human umbilical vein endothelial cells (HUVECs), they support the adhesion, proliferation, and differentiation of host cells and promote the establishment of well-organized pulp-like tissue and neovasculature [97].

Based on the laboratory studies of regeneration, it is reasonable to speculate that biological and functional regeneration of dentin–pulp complex will become feasible in the future. However, only a small quantity of research on hydrogel scaffolds has been carried out in vivo, and no clinical reports are available to date. Additionally, the lack of dentin tubular formation remains a challenge, along with difficulties in achieving temporal and orderly spatial reconstruction. Further in vivo and comparative studies are needed to expand our current understanding of pulp tissue regeneration.

### 3.2. Hydrogels for Periodontal Tissue Regeneration

Periodontitis is a well-known oral infectious disease characterized by inflammatory and the destruction of periodontal tissue, which can lead to an accelerated loss of alveolar bone and ultimately result in teeth loss [98,99]. In 2018, a new classification of periodontal disease and peri-implant disease was established at the Joint World Symposium in Chicago. The criteria for defining periodontal health are the absence of periodontitis in intact or reduced periodontal tissue, less than 10% bleeding on probes, and periodontal pockets less than or equal to 3 mm in depth. In the new classification, periodontitis previously categorized as “chronic” or “aggressive” is now reclassified as a single “periodontitis”, with “stage” and “grade” classification. Systemic diseases and the status of affected periodontal tissue diseases have been updated. Moreover, for the first time, the classification includes peri-implant diseases and states. Moreover, periodontitis has been strongly linked to systemic diseases such as coronary diseases, atherosclerotic vascular diseases, diabetes mellitus, Alzheimer’s disease, and various inflammatory comorbidities [100]. Undoubtedly, this imposes significant financial and medical burdens on patients and the government [101].

Traditionally, mechanical debridement and flap surgery aiming at removing plaque have been effective in preventing the progress of inflammation and the destruction of periodontal tissue [102]. However, the reconstruction of both the structures and functions of periodontal tissue remains a great challenge and the ideal therapeutic objective of periodontal disease. Considering the physiological structure of periodontal tissues, the morphology and functional regeneration require the simultaneous or sequential repair of three components of periodontal tissue, including the periodontal ligament (PDL), which fixes the tooth, the cementum covering the root surface, and the alveolar bone supporting the tooth [103,104]. Periodontal tissue engineering has emerged as a promising technique that combines stem cells, biological scaffolds, and growth factors to promote periodontal tissue regeneration [105].

Recently, plenty of biomaterial scaffolds have been designed to suppress inflammation while promoting the regeneration of damaged periodontal tissue simultaneously. Ozone-based antimicrobial hydrogels are a promising area of investigation. Reactive oxygen atoms produced by ozone decomposition can cause bacterial death through oxidative denaturation of bacterial proteins and irreversible bacterial destruction. Unlike antibiotics, ozone produces a broad-spectrum bactericidal effect through a physicochemical mechanism that kills bacteria quickly in only a few minutes. Furthermore, it can be released in a controlled manner to minimize damage to healthy tissues [106,107]. Scribante et al. explored the use of probiotics to regulate oral microbial balance by promoting the growth of bacteria that benefit oral health. Probiotics can also produce antimicrobial agents and bacteriocins that counteract the pathogenic effects of bacterial biofilms. In a recent clinical study, the effects of natural extract-based toothpastes and probiotic-based preparations on periodontal clinical status and glycated hemoglobin levels were evaluated in patients with type 1 diabetes mellitus. The results showed that probiotic products significantly improved patients’ probing pocket depth, plaque index, clinical attachment level, and bleeding on probing [108,109].

**Figure 4:**
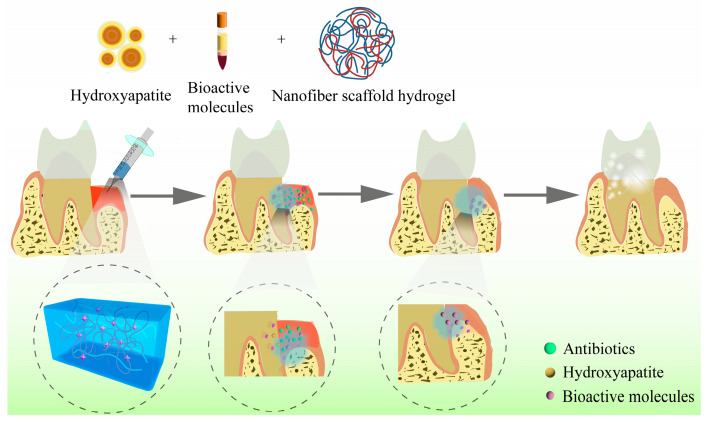
Schematic diagram of hydrogels applied in periodontal regeneration.

Any biomaterial scaffold, whether biological or synthetic, must be biocompatible and biodegradable when applied to tissue regeneration. Hydrogels are widely used as regenerative scaffolds in periodontal tissue engineering [110]. Due to their key characteristics of porosity, stiffness, and viscoelasticity, hydrogels can mimic the microenvironment of the ECM and facilitate the regulation of cell adhesion, proliferation, and osteogenic differentiation [111]. Studies have reported that when combined with drugs, stem cells, or growth factors, hydrogels exhibit outstanding potential in the complex and sophisticated process of periodontal tissue regeneration [112,113]. Figure 4 shows a schematic diagram of hydrogels applied in periodontal regeneration.

Collagen is the fundamental component of the ECM. It is composed of many specific cell signaling binding domains that facilitate cell adhesion, preserve cell phenotype, and guide cell growth, proliferation, and differentiation. For instance, Jung et al. reported that collagen hydrogels with different porosities can serve as a perfect scaffold for PDL repair and periodontal regeneration [114]. Specifically, collagen hydrogels with porosity higher than 80% significantly improve PDL cell proliferation and promote PDL-like tissue formation during a two-week period of PDL/water-soluble chitin coculture. However, collagen hydrogels often exhibit poor stability and mechanical properties and can cause immune and inflammatory reactions in animal models [115]. Therefore, investigating novel collagen sources for hydrogel is an urgent and essential task in scientific research.

HA, another important component of the ECM of connective tissues and periodontal ligament matrix, has valuable potential in periodontal tissue regeneration [116,117,118,119]. Studies have shown that HA can modulate cell adhesion, migration, and differentiation by binding proteins and cell-surface receptors. When combined and modified with other ingredients, HA hydrogels exhibit excellent mechanical properties, swelling, and low degradation speed in periodontal tissue engineering. In Babo’s study, an injectable hydrogel system was fabricated by incorporating methacrylate HA and platelet lysate (PL) to improve their mechanical properties and resilience to degradation and enhance antimicrobial activity. Furthermore, by providing ample space and stability, the hydrogel system promotes the adhesion and proliferation of hPDLFs. When inoculated on the surface of hydrogels containing 100% PL, hPDLFs migrated 70 μm deep in the hydrogel after 21 days of incubation, indicating great potential in periodontal therapy [120]. Miranda et al. created a hybrid CS–HA hydrogel scaffold for tissue engineering. In vitro cell culture tests demonstrated that the CS–HA porous structure promotes significant cell migration in periodontal tissue regeneration [121]. In a clinical study, the efficacy of a biohydrogel containing recombinant human fibroblast growth factor type 2 (rhFGF-2) in a hyaluronic acid (HA) vehicle was evaluated for treating periodontal bone intraosseous defects. The study included thirty adult patients, with the control group being treated with papilla-preserved flaps for open debridement, and the trial group applying rhFGF-2/HA topically to the intraosseous defect. After one year, the trial group showed significantly greater reductions in probing depth (PD) (5.5 vs. 2.9 mm), gains in probing attachment level (PAL) compared to the control group (4.8 vs. 2.2 mm), and shallower residual PD (4.2 vs. 6.6 mm) [122].

CS is a natural cationic polymer with a chemical structure and biological properties similar to glycosaminoglycan polysaccharide. Due to its excellent biocompatibility, biodegradability, and antimicrobial activity, CS is frequently used in periodontal therapy. Recently, Xu et al. developed an injectable and thermosensitive hydrogel system based on CS, composed of *β*-sodium glycerophosphate (*β*-GP) and gelatin, which continuously release aspirin (ASP) and erythropoietin for up to 21 days. Both in vitro and in vivo studies suggest that the CS/*β*-GP/gelatin hydrogels possess outstanding biocompatibility and remarkable anti-inflammation and periodontium regeneration ability, providing immense potential for the treatment of periodontal disease [123].

In addition to the hydrogels mentioned above, other natural hydrogels, such as gelatin and alginate, also show potential for use in novel periodontal tissue engineering. For example, gelatin/glycidyl methacrylated dextran hybrid hydrogels loaded with bone morphogenetic proteins (BMP) not only enhance the attachment, proliferation, and osteogenic differentiation of PDLCs, but also promote the regeneration of periodontal tissue [124]. Alginate hydrogels have excellent swelling ratios, degradation periods, and bovine serum albumin releasing abilities. In vivo and in vitro experiments have shown favorable biocompatibility and superior osteoinductive ability, indicating its promise as a bone regeneration material in dental clinic [125]. However, it is worth mentioning that all natural hydrogels face the obstacle of a relatively fast degradation speed as the ideal regeneration material. Therefore, modifications and combinations to improve their mechanical performance should be evaluated in the near future.

Synthetic hydrogels, such as PEG and GelMA, have exhibited outstanding mechanical properties and stability but limited biocompatibility and degradability when compared to natural hydrogels [126]. PEG is an FDA-approved hydrophilic biomaterial widely used in biomedical research due to its hydrophilicity, biocompatibility, and flexibility [127]. Fraser et al. created a PEG hydrogel with peptides to regulate two key functions of PDLC/ALP activity and matrix mineralization. In vitro experiments indicated that peptide-modified PEG hydrogels accelerated matrix cell adhesion and mineralization, while in vivo studies showed that PEG hydrogels significantly improved new bone formation [128]. GelMA, a recently developed photosensitive hydrogel biomaterial, has gained attention as a scaffold that mimics the 3D cell microenvironment. In a study by Pan et al., periodontal ligament stem cells were encapsulated in GelMA hydrogels to promote bone regeneration. The highly porous and interconnected microstructure of GelMA hydrogels not only provided an optimal microenvironment for proliferation, migration, and osteogenic differentiation of stem cells, but also encouraged significant alveolar bone regeneration in an in vivo rat model [129].

Hydrogels are renowned in biomedical research for their prominent biocompatibility and biodegradability, making them popular as biomaterial scaffolds and bioactive ingredient delivery systems. Both natural and synthetic hydrogels provide an optimal niche for mesenchymal stem cell adhesion, proliferation, migration, and differentiation, while also modulating inflammatory reactions and regulating the immune microenvironment to promote structural and functional regeneration of periodontal tissue. However, mechanical properties and biological profiles pose an obstacle in achieving the desired regenerative outcomes, and much work still needs to be done to address these challenges in the near future.

### 3.3. Hydrogels for Mandible Regeneration

The mandible is a crucial component of the human face, as it plays a vital role in mastication, pronunciation, and speaking, while also contributing to facial contour and shape [130]. However, loss of bone tissue in the oral cavity can occur due to various reasons, such as traumas, tumors, infections, functional atrophy, congenital disorders, and periodontitis, leading to different degrees of impact on the patient’s facial appearance and oral function [131,132,133,134]. Certain drugs, such as isophosphonates, can also lead to jaw osteonecrosis, known as drug-related osteonecrosis of the jaw (MRONJ), as they are anti-angiogenic or anti-osteoporotic [135]. Even a short-term discontinuation of such drugs does not eliminate their effects on the jaws, and segmental resection becomes the only solution to remove necrotic bones in the late stage of MRONJ [136]. Although bones can heal themselves, repairing bone defects larger than a critical size and ununited fractures remains a challenge in clinical practice [137]. Moreover, self-repair is not feasible when the loss of the mandible exceeds 10% [138]. Thus, there is a pressing need for an effective method to promote local osteogenesis of the mandible [139]. Currently, mandibular defects are treated through various methods such as traction osteogenesis, metal implants, and bone grafting. The fibular free flap graft is considered the gold standard for mandibular reconstruction [140]. However, the limited availability of bone and donor site morbidity are the primary limitations of this technique [141]. Therefore, it is crucial to develop a bone regeneration strategy that does not require autograft.

In recent years, biomaterials for bone tissue engineering have progressed rapidly. However, mandibular repair is a particularly challenging clinical situation due to several factors, including a microbial-rich environment, lack of soft tissue coverage, ongoing adjuvant radiotherapy requirements, complex geometry, specific anatomy (teeth), high stress axial, and non-axial (cantilever) loading. Mandibular lesions associated with oral cancer are typically 6 to 10 cm in length and may take several months to produce bone, making the mid-term and long-term mechanical properties of the scaffold critical for significant proportions of bone defects [142]. The aim of oromandibular reconstruction is to restore both function and shape. Therefore, techniques for repairing oromandibular bone require more control over the generated shape than the musculoskeletal system [143]. Hydrogel scaffolds have several advantages for jaw reconstruction, including the following:

(1) They are soft and rubbery, which reduces the inflammatory response in the tissues and cells surrounding them [144];
(2) Their porous structures are similar to the ECM, facilitating cell adhesion and proliferation [145];
(3) They are ideal for delivering bioactive factors, antimicrobial agents, and nanoparticles to enhance therapeutic effects such as osteogenesis, angiogenesis, and anti-infection;
(4) They have controlled degradability [146] with rapid growth of the mandible, which eliminates the need for a second excisional procedure;
(5) Their injectable nature allows for minimally invasive surgery to meet aesthetic restoration goals of facial features;
(6) They can increase structural stability and mechanical strength through various methods.

**Figure 5:**
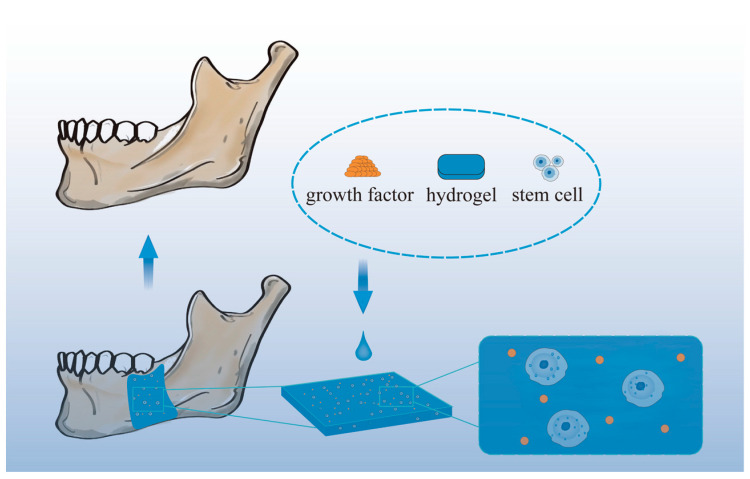
Schematic diagram of mandibular restoration using hydrogel biomaterials.

Figure 5 shows a schematic diagram of mandibular restoration using hydrogel biomaterials.

Hydrogel systems play a significant role in the treatment of infected jaw defects by facilitating antibiotic release. For instance, Sun et al. prepared vancomycin hydrochloride (Van)-SBA-15 using mesoporous silica (SBA-15) and encapsulated Van in a CS–sodium glycerophosphate–sodium alginate hydrogel. The hydrogel enabled the sustained release of Van and holds potential for the treatment of infected jaw defects [147]. In another study, clindamycin (CDM) was shown to penetrate human bone tissue effectively [148]. Sungkhaphan et al. developed a biodegradable composite hydrogel consisting of carboxymethyl CS and CDM-loaded mesoporous silica nanoparticles (MCM-41) with dual antibacterial activity and osteogenic potency. The hydrogel loaded with CDM maintained antibacterial activity against Streptococcus sanguis for at least 14 days in vitro [149]. Smart pH-responsive hydrogel scaffolds can serve as effective carriers for the local release of antimicrobial agents. Quaternary pyridinium salts (QPS), which are pH-sensitive molecules, are incorporated into the scaffold. In the presence of inflammatory conditions (i.e., acidic mediators), the QPS triggers the release of antimicrobial silver nanoparticles (AgNPs). This mechanism promotes tissue healing and reduces drug resistance [150]. In addition to loading antibiotics, researchers have also constructed the surface microstructure of hydrogels to prevent bacterial adhesion or achieve photothermal sterilization, thereby preventing infection [151,152,153].

Furthermore, hydrogels have been used to mimic native bone and enhance bone regeneration. For example, Kumar et al. developed a bone tissue engineering biphasic construct loaded with bone BMP-2. They used a gelatin–HA hydrogel to bind to the osteogenic cue BMP-2, which was then loaded onto a PCL scaffold. The construct mimicked native bone and consisted of cortical bone and cancellous bone for vertical jawbone augmentation. In vitro studies showed that the cell viability of BMP-2 was maintained in the hydrogel for 21 days, and bone markers increased on the third and fourteenth days [154]. Vaquette et al. also conducted similar work in a vertical regeneration of jawbone, where they evaluated the bone formation capacity and dimensional stability of biphasic scaffolds fabricated with BMP-2 functionalized additives, which promoted dimensionally stable bone regeneration to support dental implant osseointegration [155]. To address the mechanical limitations of natural hydrogels in load-bearing areas, researchers have turned to synthetic hydrogels due to their more stable mechanical properties. For example, Suo et al. prepared graphene oxide (GO)/HA/CS viscoelastic composite scaffolds using low-temperature 3D printing technology to control the shape of the material to match it better to the shape of the jawbone. The addition of GO improved the poor mechanical properties of the hydrogel and added certain antibacterial properties [156]. Natural hydrogels are often used in tissue engineering due to their better biocompatibility and degradability. However, they have limitations in terms of mechanical strength and stability, which make them unsuitable for load-bearing areas. Synthetic hydrogels, on the other hand, can be designed to have specific mechanical properties and are often used in load-bearing applications. Lei et al. developed an injectable, thermoresponsive hydrogel for local and long-term codelivery of microRNA-222 and aspirin. The hydrogel is structured as a core–shell embedded with mesoporous silica nanoparticles and is composed of poly(ethylene glycol)-b-poly(lactic acid-ethanolic acid)-b-poly(N-isopropylacrylamide) [157]. Mesenchymal stem cells (MSCs), which can differentiate into various cell types, including bone tissue [158], are often combined with hydrogels for jawbone repair [159]. In a clinical study, 37 patients with possible jaw defects were recruited and treated with implants. The effect of PEG hydrogel membranes on vertical bone filling was evaluated. the test group received a PEG hydrogel membrane while the control group received a collagen membrane. After a six-month healing period, surgical repositioning was performed to assess the change in vertical bone height from baseline. The mean vertical defect fill was 5.63 ± 1.84 mm and 4.25 ± 1.16 mm for the test and control groups, respectively, with success rates of 94.9% and 96.4% for the test and control groups, respectively. The results indicate that PEG hydrogel membranes are as effective as collagen membranes in terms of treatment efficacy [160].

To address mandibular abnormalities in different pathogenic conditions, the ideal hydrogel should possess improved mechanical properties, antimicrobial capabilities, an injectable form for minimally invasive surgeries, or customizable 3D bioprinting capability. Advanced hydrogel technology can potentially provide a more personalized and precise approach to challenging mandibular repair.

### 3.4. Hydrogels for Soft Tissue Healing

**Figure 6:**
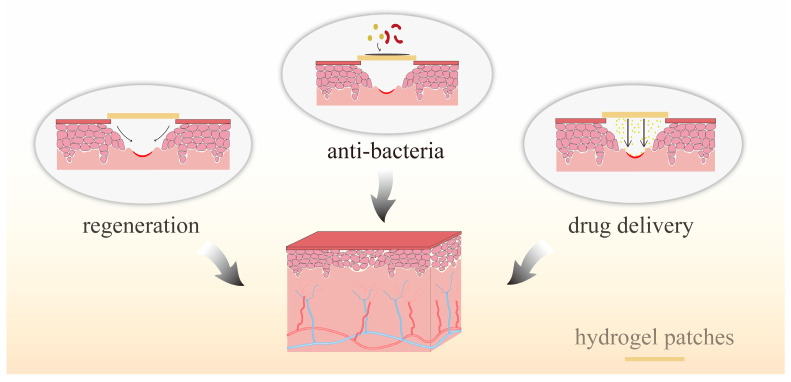
Role of hydrogel patches in treating oral mucosal diseases.

While dental and skeletal repair are the primary focus of craniofacial applications, the need for soft tissue regeneration is equally crucial [161]. Oral and maxillofacial soft tissues include various structures such as periodontal tissues, tongue, oral mucosa, muscles, skin. Soft tissue regeneration remains a major challenge in contemporary medicine and dentistry. Periodontitis, gingival recession, and chronic inflammation of the gums can lead to tooth loss. In the cases of long-term tooth loss, proper dental restoration and aesthetic outcomes heavily depend on critical soft tissues [162]. After dental implantation, the bacterial environment in the oral cavity can cause inflammation around the implant. Enhanced sealing of the soft tissues around the implant can improve the success of the implant [163]. The application of hydrogels on periodontal tissues has been described in Section 3.2 of this review, while this section will focus on the use of hydrogels in treating oral mucosal diseases. Infection is the most common preventable challenge in wound healing [164]. Hydrogel wound dressings can play a vital role in wound healing by protecting the wound from contamination and trauma and creating an optimal environment to support endogenous cell growth and promote wound closure [165]. Due to their high-water content and similarity to biological soft tissues, hydrogels can be seamlessly integrated into the human biological environment [166]. Figure 6 illustrates the role of hydrogel patches in treating oral mucosal diseases.

Soft tissue engineering has emerged as a new approach to repair damaged or diseased soft tissues and organs [167]. Hydrogels, which are ideal biomaterials to mimic soft tissues [168], have been developed for this purpose. However, treating oral diseases can be challenging because high doses of topical medications are often required, and systemic administration is typically the main treatment option for small lesions of the oral mucosa. The moist, dynamic, and unstable environment of the oral cavity, including constant salivary flushing, and exposure to microorganisms, enzymes, and food and beverages, makes it difficult to maintain wound dressings. Furthermore, hydrogels must meet high demands for structural and mechanical stability [169]. One way to improve the mechanical properties of hydrogels is to construct hybrid hydrogels, such as fiber-reinforced hydrogels [170], bilayer network hydrogels [171,172], and semipenetrating hydrogels [173]. While several topical treatments using gels or creams are currently available in the market, such as Bayer oral ulcer gel [174], the accidental swallowing of the delivery system and continuous dilution of saliva may lead to low residence time of the formulation in the oral cavity, resulting in low bioavailability of the drug [175]. Materials used to treat oral mucosal wounds must also possess adhesion and antimicrobial properties [176]. In addition, biodegradability and biocompatibility are required, and the toxicity of the material once it enters the gastrointestinal tract must be considered.

Zheng et al. chemically crosslinked chitosan/fucose gum (CF) composite hydrogels containing trimethoprim (TA). The addition of fucoidan significantly improved the swelling behavior, mechanical strength, and adhesive quality of CS hydrogels. The composite hydrogel showed a nearly 600% swelling rate in artificial saliva. The CF hydrogel exhibited a higher shear strength (63.4 ± 7.60 kPa) than the CS hydrogel (25.17 ± 6.60 kPa), making it better suited for adhering to oral mucosa. In addition, the TA added to CF hydrogels enhanced their elastic properties, reduced inflammatory responses, and promoted the development of mature, well-organized collagen fibers. The developed composite hydrogels demonstrated potent antimicrobial, cytocompatible, and histocompatible properties, making them a potential treatment for oral inflammation by creating oral mucosal patches [177].

**Figure 7:**
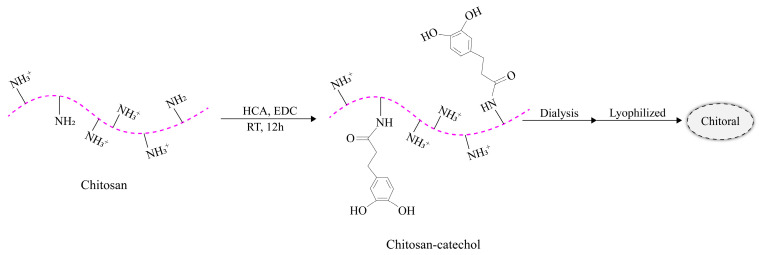
Synthesis mechanism of Chitoral.

Gingival mesenchymal stem cells (GMSCs) were enclosed in a hydrogel made of alginate and GelMA. The GMSCs enclosed in hydrogels can accelerate wound healing, soft tissue regeneration, and promote collagen deposition [178]. Yi et al. developed a bioink consisting of injectable platelet-rich fibrin, alginate, and gelatin that can be shaped according to individual patient needs [179]. Zhang et al. designed a photosensitive cyclic o-nitrobenzyl-modified HA, choosing high molecular weight HA as the backbone of the gel due to its biocompatible and anti-inflammatory properties. Application tests in oral mucosal defects in rats and pigs showed that the gel was effective in protecting the damaged site from the complex oral environment for more than 24 h while promoting oral mucosal wound healing [180]. Qi et al. presented a photosensitive hydrogel synthesized from CS and the photocrosslinker Ru bipyridine. After visible light irradiation, the dermal aldehyde group was shed, and Ru bipyridine acted as a bactericidal agent in a humid environment. Furthermore, this photoreactive hydrogel can be considered as a good stem cell presentation system. Because the material is not lethal under dark conditions, its 3D structure facilitates the growth and sustained release of bone marrow-derived mesenchymal stem cells (BMSCs). This light-responsive antimicrobial hydrogel capable of presenting BMSCs has promising applications in the wound repair of oral mucosa [181]. Kim et al. prepared artificial oral mucosal tissue models using GelMA hydrogels cocultured with human gingival fibroblasts (HGFs) and human oral keratinocytes (HOKs). After 14 days of culture on the surface of the hydrogel, the number of HOK cells increased and showed continuous cell proliferation. Histological evaluation showed that bilayer GelMA hydrogels of HGFs and HOKs could be used to develop artificial oral mucosal tissues [182]. Wang et al. designed a dry polyacrylic acid–CS–aminolevulinic acid (ALA) interpenetrating network hydrogel (PACA) patch with high adhesion strength. The PACA patch rapidly and stably adhered to moist oral mucosa and delivered 5-ALA [183]. To address the challenges in oral drug delivery, Ryu et al. performed in situ crosslinking and curing of mucoadhesive polymer hydrogels. They developed a CS–catechol mucoadhesive patch called “Chitoral,” and demonstrated its therapeutic efficacy in treating oral mucositis using tretinoin as a model drug [184]. Figure 7 illustrates the synthesis mechanism of Chitoral.

In a clinical study, the effectiveness of topical oral Omega-3 hydrogel for the prevention of radiation-induced oral mucositis was evaluated. Patients in the test group were treated with topical oral Omega-3 hydrogel, while patients in the control group were treated with conventional methods. The severity of oral mucositis was assessed as the final outcome within a timeframe of up to 6 weeks [185].

Hydrogels are attractive for use in oral mucosa applications due to their adjustable modulus, which enables them to conform well to the irregular surface of the oral mucosa. However, the oral cavity is a complex and bacteria-rich environment, and the high moisture content of the oral mucosa makes it challenging to maintain the adhesion properties of hydrogel patches. Therefore, improving the adhesion properties and environmental stability of hydrogel dressings is an important area of research in this field.

## 4. Clinical Applications of Hydrogels in Oral Tissue Repair and Regeneration

There have been several clinical studies on the use of hydrogels for repairing oral tissues, and information about these studies is listed in Table 1. Most of these studies focused on the use of hydrogels for treating oral mucositis and periodontitis, with one study investigating the treatment of oral bacterial infections and another exploring tooth loss.

## 5. Conclusions and Future Perspectives

Oral health is an integral part of general health and quality of life. Infections, genetic diseases, cancer, and trauma can lead to tissue defects in dental, oral, and craniofacial structures. In recent years, numerous research works have been conducted in biomaterials for dentistry to improve clinical outcomes and quality of life for patients.

Hydrogels are emerging as promising dental biomaterials due to their high affinity for water, allowing them to absorb large amounts of water or biological fluids while remaining insoluble. Moreover, hydrogels allow for exceptional fusion with surrounding tissues, reducing the possibility of inflammatory reactions. Furthermore, hydrogels are available in various forms, including natural and synthetic, making them versatile. Hydrogels offer an alternative to root canal therapy in pulp tissue regeneration. In periodontal therapy, hydrogels can serve as an antimicrobial topical delivery system and promote periodontal tissue regeneration. Hydrogels are also flexible and can be used in conjunction with other biomaterials to create scaffolds with improved mechanical properties. Additionally, they can carry bioactive molecules to enhance bone tissue regeneration. The regeneration of low modulus tissues, below 10 kPa, remains an unmet need in tissue engineering, as these tissues often serve irreplaceable functions. However, the adjustable modulus range of soft hydrogels provides the potential for regenerating oral soft tissues. The crucial role of hydrogels in drug delivery cannot be overstated. The oral cavity harbors numerous and complex microorganisms, and many oral problems are caused by an unbalanced microbial environment. Therefore, regulating the microbial balance in the oral cavity and fighting against bacterial infections are crucial for effective oral diseases management. In addition to their effectiveness in delivering antibiotics, hydrogels can be combined with novel antimicrobial approaches, such as ozone-based hydrogels, used in combination with probiotics. Furthermore, recently developed stimuli-responsive hydrogels can produce antimicrobial effects in response to external stimuli, such as light and pH.

Although hydrogels offer many advantages as biomaterials, there are still several challenges that need to be addressed. For example, the mechanical properties of most hydrogels are poor and their stability needs to be improved. In the context of mandibular bone regeneration and repair, addressing how the hydrogel scaffold can meet the mechanical properties required for the load-bearing site remains a challenge that needs further investigation. Similarly, improving the adhesion of hydrogel patches to oral mucosa is a popular topic in the application of oral mucosal drug delivery and wound healing. In addition, controlling the degradation rate of hydrogels is critical for drug or cell delivery systems to ensure effective anti-infection and tissue repair. It is important to ensure that the drug reaches the effective concentration within a specific timeframe. While many current studies focus on in vitro or animal models, further research is needed to bridge the gap between preclinical studies and clinical applications.

Currently, there is a limited number of studies on the application of hydrogels in dentistry. However, over the past five years, there has been an increasing trend in research, with many studies demonstrating excellent results. Although many studies have been conducted on animals, there are only a few clinical trials evaluating the effects of hydrogel use in oral clinical applications. To accurately assess the benefits of hydrogels in human patients, more clinical trials are necessary. Based on the literature reviewed, hydrogels appear to be promising biomaterials in dentistry and deserve further attention in biomedical research to address current challenges and limitations.

## Figures and Tables

**Table 1:** Clinical applications of hydrogels in oral tissue repair and regeneration [185].

Status	Study Title	Conditions	Interventions
Completed	Omega-3 hydrogel and prevention of oral mucositis	Mucositis oral	Drug: topical oral Omega-3 hydrogel; drug: conventional preventive treatment
Not yet recruiting	Efficacy of EGF-loaded self-healing gel in treating oral mucositis	Oral mucositis	Drug: EGF-loaded hydrogel; drug: hydrogel
Completed	A study to evaluate efficacy of MuGard for amelioration of oral mucositis in head and neck cancer patients	Oral mucositis	Device: MuGard; device: control rinse
Recruiting	MucoLox formulation to mitigate mucositis symptoms in head/neck cancer	Mucositis oral head and neck cancer	Other: MucoLox; other: sodium bicarbonate
Unknown	Topical chamomile in preventing chemotherapy-induced oral mucositis	Oral mucositis due to chemotherapy	Drug: chamomile topical oral gel; drug: miconazole topical gel; drug: BBC oral spray; drug: oracure gel
Completed	Impact of daily use of emanate tray adjunct to full mouth debridement compared to full mouth debridement alone	Wound heal mouth; wound periodontal inflammation	Device: emanate tray
Completed	Nitazoxanide as a new local adjunctive to nonsurgical treatment of moderate periodontitis	Periodontitis	Procedure: scaling and root planing; drug: nitazoxanide hydrogel
Completed	Efficacy of proanthocyanidins in nonsurgical periodontal therapy	Periodontitis, adult	Procedure: minimally invasive nonsurgical therapy; combination product: subgingival application of collagen hydrogels with proanthocyanidins; diagnostic test: collection of saliva samples
Completed	Does hyaluronic acid affect periodontal treatment?	Periodontitis	Procedure: scaling and root planing; drug: hyaluronic acid gel (HA) and SRP; drug: HA mouthrinse and SRP; drug: HA mouthrinse + gel and SRP
Completed	Use of adhesion molecule-loaded hydrogel with minimally invasive surgical technique in treating periodontal intrabony defects	Periodontitis	Drug: RGD peptide
Completed	PLGA nanoparticles entrapping ciprofloxacin to treat E-Fecalis infections in endodontics	Bacterial infections oral	Device: chitosan-coated PLGA nanoparticles entrapping ciprofloxacin incorporated in smart gels; device: ciprofloxacin paste and solution
Unknown	Hyaluronic acid effect on xenogenic bone healing	Bone resorption tooth loss	Procedure: ridge preservation: tooth extraction and immediate bone grafting in the socket

## Data Availability

Not applicable.

## References

[1] Liang J., Peng X., Zhou X., Zou J., Cheng L. (2020). Emerging Applications of Drug Delivery Systems in Oral Infectious Diseases Prevention and Treatment. Molecules.

[2] Lamont R.J., Koo H., Hajishengallis G. (2018). The Oral Microbiota: Dynamic Communities and Host Interactions. Nat. Rev. Microbiol..

[3] Fukushima-Nakayama Y., Ono T., Hayashi M., Inoue M., Wake H., Ono T., Nakashima T. (2017). Reduced Mastication Impairs Memory Function. J. Dent. Res..

[4] Gao H., Wu N., Wang N., Li J., Sun J., Peng Q. (2022). Chitosan-Based Therapeutic Systems and Their Potentials in Treatment of Oral Diseases. Int. J. Biol. Macromol..

[5] Ye S., Wei B., Zeng L. (2022). Advances on Hydrogels for Oral Science Research. Gels.

[6] Peres M.A., Macpherson L.M., Weyant R.J., Daly B., Venturelli R., Mathur M.R., Listl S., Celeste R.K., Guarnizo-Herreño C.C., Kearns C. (2019). Oral Diseases: A Global Public Health Challenge. Lancet.

[7] Fischer N.G., Münchow E.A., Tamerler C., Bottino M.C., Aparicio C. (2020). Harnessing Biomolecules for Bioinspired Dental Biomaterials. J. Mater. Chem. B.

[8] Şenel S., Özdoğan A.I., Akca G. (2021). Current Status and Future of Delivery Systems for Prevention and Treatment of Infections in the Oral Cavity. Drug Deliv. Transl. Res..

[9] Yang J., Liu F., Zhou C., Li H., Yang G., Fang S., Lee I.S., Liu Y., Bai H., Chen C. (2023). 3D Printed Porous Titanium Filled with Mineralized UV-Responsive Chitosan Hydrogel Promotes Cell Proliferation and Osteogenesis in vitro. J. Mater. Sci. Technol..

[10] Hu C., Zhang M., Wu J., Cao X., Chen L., Yan J., Liang G., Tan J. (2023). Bisphosphonate-Modified Functional Supramolecular Hydrogel Promotes Periodontal Bone Regeneration by Osteoclast Inhibition. ACS Appl. Mater. Interfaces.

[11] Deng H., Dong A., Song J., Chen X. (2019). Injectable Thermosensitive Hydrogel Systems Based on Functional PEG/PCL Block Polymer for Local Drug Delivery. J. Control. Release.

[12] Wang Y., Zhang W., Gong X., Zhao C., Liu Y., Zhang C. (2023). Construction of Carboxymethyl Chitosan Hydrogel with Multiple Cross-Linking Networks for Electronic Devices at Low Temperature. ACS Biomater. Sci. Eng..

[13] Yang Q., Peng J., Xiao H., Xu X., Qian Z. (2022). Polysaccharide Hydrogels: Functionalization, Construction and Served as Scaffold for Tissue Engineering. Carbohydr. Polym..

[14] Wang K., Han Z. (2017). Injectable Hydrogels for Ophthalmic Applications. J. Control. Release.

[15] Yin Y., Hu B., Yuan X., Cai L., Gao H., Yang Q. (2020). Nanogel: A Versatile Nano-Delivery System for Biomedical Applications. Pharmaceutics.

[16] Tavelli L., McGuire M.K., Zucchelli G., Rasperini G., Feinberg S.E., Wang H.L., Giannobile W.V. (2020). Extracellular Matrix-Based Scaffolding Technologies for Periodontal and Peri-Implant Soft Tissue Regeneration. J. Periodontol..

[17] Hussey G.S., Dziki J.L., Badylak S.F. (2018). Extracellular Matrix-Based Materials for Regenerative Medicine. Nat. Rev. Mater..

[18] Tong X., Yang F. (2018). Recent Progress in Developing Injectable Matrices for Enhancing Cell Delivery and Tissue Regeneration. Adv. Healthc. Mater..

[19] Alavi S.E., Panah N., Page F., Gholami M., Dastfal A., Sharma L.A., Shahmabadi H.E. (2022). Hydrogel-Based Therapeutic Coatings for Dental Implants. Eur. Polym. J..

[20] Ansari S., Seagroves J.T., Chen C., Shah K., Aghaloo T., Wu B.M., Bencharit S., Moshaverinia A. (2017). Dental and Orofacial Mesenchymal Stem Cells in Craniofacial Regeneration: The Prosthodontist’s Point of View. J. Prosthet. Dent..

[21] Skudutyte-Rysstad R., Eriksen H. (2006). Endodontic Status amongst 35-Year-Old Oslo Citizens and Changes over a 30-Year Period. Int. Endod. J..

[22] Riba H., Al-Zahrani S., Al-Buqmi N., Al-Jundi A. (2017). A Review of Behavior Evaluation Scales in Pediatric Dentistry and Suggested Modification to the Frankl Scale. EC Dent. Sci..

[23] Tibúrcio-Machado C., Michelon C., Zanatta F., Gomes M.S., Marin J.A., Bier C.A. (2021). The Global Prevalence of Apical Periodontitis: A Systematic Review and Meta-Analysis. Int. Endod. J..

[24] Frencken J.E., Sharma P., Stenhouse L., Green D., Laverty D., Dietrich T. (2017). Global Epidemiology of Dental Caries and Severe Periodontitis–a Comprehensive Review. J. Clin. Periodontol..

[25] Colley H., Said Z., Santocildes-Romero M., Baker S., D’Apice K., Hansen J., Madsen L.S., Thornhill M., Hatton P., Murdoch C. (2018). Pre-Clinical Evaluation of Novel Mucoadhesive Bilayer Patches for Local Delivery of Clobetasol-17-Propionate to the Oral Mucosa. Biomaterials.

[26] Dudding T., Haworth S., Lind P.A., Sathirapongsasuti J.F., Tung J.Y., Mitchell R., Colodro-Conde L., Medland S.E., Gordon S., Timpson N.J. (2019). Genome Wide Analysis for Mouth Ulcers Identifies Associations at Immune Regulatory Loci. Nat. Commun..

[27] Piva E., Silva A.F., Nor J.E. (2014). Functionalized Scaffolds to Control Dental Pulp Stem Cell Fate. J. Endod..

[28] León-López A., Morales-Peñaloza A., Martínez-Juárez V.M., Vargas-Torres A., Zeugolis D.I., Aguirre-Álvarez G. (2019). Hydrolyzed Collagen—Sources and Applications. Molecules.

[29] Fallacara A., Baldini E., Manfredini S., Vertuani S. (2018). Hyaluronic Acid in the Third Millennium. Polymers.

[30] Reakasame S., Boccaccini A.R. (2018). Oxidized Alginate-Based Hydrogels for Tissue Engineering Applications: A Review. Biomacromolecules.

[31] Seliktar D. (2012). Designing Cell-Compatible Hydrogels for Biomedical Applications. Science.

[32] Cao H., Duan L., Zhang Y., Cao J., Zhang K. (2021). Current Hydrogel Advances in Physicochemical and Biological Response-Driven Biomedical Application Diversity. Signal Transduct. Target. Ther..

[33] Nie L., Li X., Chang P., Liu S., Wei Q., Guo Q., Wu Q., Fan L., Okoro O.V., Shavandi A. (2022). A Fast Method for in Vitro Biomineralization of PVA/Alginate/Biphasic Calcium Phosphate Hydrogel. Mater. Lett..

[34] Leucht A., Volz A.C., Rogal J., Borchers K., Kluger P. (2020). Advanced Gelatin-Based Vascularization Bioinks for Extrusion-Based Bioprinting of Vascularized Bone Equivalents. Sci. Rep..

[35] Ikeda Y., Holcroft J., Ikeda E., Ganss B. (2022). Amelotin Promotes Mineralization and Adhesion in Collagen-Based Systems. Cell. Mol. Bioeng..

[36] Huang Q., Huang X., Gu L. (2021). Periodontal Bifunctional Biomaterials: Progress and Perspectives. Materials.

[37] Andonegi M., Las Heras K., Santos-Vizcaíno E., Igartua M., Hernandez R.M., de la Caba K., Guerrero P. (2020). Structure-Properties Relationship of Chitosan/Collagen Films with Potential for Biomedical Applications. Carbohydr. Polym..

[38] Xie X., Li X., Lei J., Zhao X., Lyu Y., Mu C., Li D., Ge L., Xu Y. (2020). Oxidized Starch Cross-Linked Porous Collagen-Based Hydrogel for Spontaneous Agglomeration Growth of Adipose-Derived Stem Cells. Mater. Sci. Eng. C.

[39] Tabatabaei F., Moharamzadeh K., Tayebi L. (2020). Fibroblast Encapsulation in Gelatin Methacryloyl (GelMA) versus Collagen Hydrogel as Substrates for Oral Mucosa Tissue Engineering. J. Oral Biol. Craniofacial Res..

[40] Kwon M.Y., Wang C., Galarraga J.H., Puré E., Han L., Burdick J.A. (2019). Influence of Hyaluronic Acid Modification on CD44 Binding towards the Design of Hydrogel Biomaterials. Biomaterials.

[41] Passi A., Vigetti D. (2019). Hyaluronan as Tunable Drug Delivery System. Adv. Drug Deliv. Rev..

[42] Chircov C., Grumezescu A.M., Bejenaru L.E. (2018). Hyaluronic Acid-Based Scaffolds for Tissue Engineering. Rom. J. Morphol. Embryol..

[43] Graça M.F., Miguel S.P., Cabral C.S., Correia I.J. (2020). Hyaluronic Acid—Based Wound Dressings: A Review. Carbohydr. Polym..

[44] Singh H., Rathee K., Kaur A., Miglani N. (2021). Pulp Regeneration in an Immature Maxillary Central Incisor Using Hyaluronic Acid Hydrogel. Contemp. Clin. Dent..

[45] Abbass M.M., El-Rashidy A.A., Sadek K.M., Moshy S.E., Radwan I.A., Rady D., Dörfer C.E., Fawzy El-Sayed K.M. (2020). Hydrogels and Dentin–Pulp Complex Regeneration: From the Benchtop to Clinical Translation. Polymers.

[46] Parthiban S.P., He W., Monteiro N., Athirasala A., França C.M., Bertassoni L.E. (2020). Engineering Pericyte-Supported Microvascular Capillaries in Cell-Laden Hydrogels Using Stem Cells from the Bone Marrow, Dental Pulp and Dental Apical Papilla. Sci. Rep..

[47] Wang Q.Q., Liu Y., Zhang C.J., Zhang C., Zhu P. (2019). Alginate/Gelatin Blended Hydrogel Fibers Cross-Linked by Ca^2+^ and Oxidized Starch: Preparation and Properties. Mater. Sci. Eng. C.

[48] Mao Q., Hoffmann O., Yu K., Lu F., Lan G., Dai F., Shang S., Xie R. (2020). Self-Contracting Oxidized Starch/Gelatin Hydrogel for Noninvasive Wound Closure and Wound Healing. Mater. Des..

[49] Athirasala A., Lins F., Tahayeri A., Hinds M., Smith A.J., Sedgley C., Ferracane J., Bertassoni L.E. (2017). A Novel Strategy to Engineer Pre-Vascularized Full-Length Dental Pulp-like Tissue Constructs. Sci. Rep..

[50] Park J.H., Gillispie G.J., Copus J.S., Zhang W., Atala A., Yoo J.J., Yelick P.C., Lee S.J. (2020). The Effect of BMP-Mimetic Peptide Tethering Bioinks on the Differentiation of Dental Pulp Stem Cells (DPSCs) in 3D Bioprinted Dental Constructs. Biofabrication.

[51] Monteiro N., Thrivikraman G., Athirasala A., Tahayeri A., França C.M., Ferracane J.L., Bertassoni L.E. (2018). Photopolymerization of Cell-Laden Gelatin Methacryloyl Hydrogels Using a Dental Curing Light for Regenerative Dentistry. Dent. Mater..

[52] Han L., Xu J., Lu X., Gan D., Wang Z., Wang K., Zhang H., Yuan H., Weng J. (2017). Biohybrid Methacrylated Gelatin/Polyacrylamide Hydrogels for Cartilage Repair. J. Mater. Chem. B.

[53] He Y., Zhao W., Dong Z., Ji Y., Li M., Hao Y., Zhang D., Yuan C., Deng J., Zhao P. (2021). A Biodegradable Antibacterial Alginate/Carboxymethyl Chitosan/Kangfuxin Sponges for Promoting Blood Coagulation and Full-Thickness Wound Healing. Int. J. Biol. Macromol..

[54] Abasalizadeh F., Moghaddam S.V., Alizadeh E., Akbari E., Kashani E., Fazljou S.M.B., Torbati M., Akbarzadeh A. (2020). Alginate-Based Hydrogels as Drug Delivery Vehicles in Cancer Treatment and Their Applications in Wound Dressing and 3D Bioprinting. J. Biol. Eng..

[55] Pan Y., Zhao Y., Kuang R., Liu H., Sun D., Mao T., Jiang K., Yang X., Watanabe N., Mayo K.H. (2020). Injectable Hydrogel-Loaded Nano-Hydroxyapatite That Improves Bone Regeneration and Alveolar Ridge Promotion. Mater. Sci. Eng. C.

[56] Sancilio S., Gallorini M., Di Nisio C., Marsich E., Di Pietro R., Schweikl H., Cataldi A. (2018). Alginate/Hydroxyapatite-Based Nanocomposite Scaffolds for Bone Tissue Engineering Improve Dental Pulp Biomineralization and Differentiation. Stem Cells Int..

[57] Xiong X., Xiao W., Zhou S., Cui R., Xu H.H., Qu S. (2021). Enhanced Proliferation and Angiogenic Phenotype of Endothelial Cells via Negatively-Charged Alginate and Chondroitin Sulfate Microsphere Hydrogels. Biomed. Mater..

[58] Kou S.G., Peters L.M., Mucalo M.R. (2021). Chitosan: A Review of Sources and Preparation Methods. Int. J. Biol. Macromol..

[59] Saeedi M., Vahidi O., Moghbeli M., Ahmadi S., Asadnia M., Akhavan O., Seidi F., Rabiee M., Saeb M.R., Webster T.J. (2022). Customizing Nano-Chitosan for Sustainable Drug Delivery. J. Control. Release.

[60] Fabiano A., Beconcini D., Migone C., Piras A.M., Zambito Y. (2020). Quaternary Ammonium Chitosans: The Importance of the Positive Fixed Charge of the Drug Delivery Systems. Int. J. Mol. Sci..

[61] Iftime M.M., Ailiesei G.L., Ungureanu E., Marin L. (2019). Designing Chitosan Based Eco-Friendly Multifunctional Soil Conditioner Systems with Urea Controlled Release and Water Retention. Carbohydr. Polym..

[62] Wang R., Li N., Jiang B., Li J., Hong W., Jiao T. (2021). Facile Preparation of Agar/Polyvinyl Alcohol-Based Triple-Network Composite Hydrogels with Excellent Mechanical Performances. Colloids Surf. Physicochem. Eng. Asp..

[63] Munim S.A., Raza Z.A. (2019). Poly (Lactic Acid) Based Hydrogels: Formation, Characteristics and Biomedical Applications. J. Porous Mater..

[64] Bhattarai N., Gunn J., Zhang M. (2010). Chitosan-Based Hydrogels for Controlled, Localized Drug Delivery. Adv. Drug Deliv. Rev..

[65] Ganji F., Abdekhodaie M.J. (2010). Chitosan–g–PLGA Copolymer as a Thermosensitive Membrane. Carbohydr. Polym..

[66] Zou H., Wang G., Song F., Shi X. (2017). Investigation of Human Dental Pulp Cells on a Potential Injectable Poly (Lactic–Co–Glycolic Acid) Microsphere Scaffold. J. Endod..

[67] Sood S., Gupta V.K., Agarwal S., Dev K., Pathania D. (2017). Controlled Release of Antibiotic Amoxicillin Drug Using Carboxymethyl Cellulose-Cl-Poly (Lactic Acid-Co-Itaconic Acid) Hydrogel. Int. J. Biol. Macromol..

[68] Bekhouche M., Bolon M., Charriaud F., Lamrayah M., Da Costa D., Primard C., Costantini A., Pasdeloup M., Gobert S., Mallein-Gerin F. (2020). Development of an Antibacterial Nanocomposite Hydrogel for Human Dental Pulp Engineering. J. Mater. Chem. B.

[69] Fu Y., Ding Y., Zhang L., Zhang Y., Liu J., Yu P. (2021). Poly Ethylene Glycol (PEG)-Related Controllable and Sustainable Antidiabetic Drug Delivery Systems. Eur. J. Med. Chem..

[70] Chow A., Stuckey D.J., Kidher E., Rocco M., Jabbour R.J., Mansfield C.A., Darzi A., Harding S.E., Stevens M.M., Athanasiou T. (2017). Human induced Pluripotent Stem Cell-Derived Cardiomyocyte Encapsulating Bioactive Hydrogels Improve Rat Heart Function Post Myocardial Infarction. Stem Cell Rep..

[71] Ma Y., Ji Y., Zhong T., Wan W., Yang Q., Li A., Zhang X., Lin M. (2017). Bioprinting-Based PDLSC-ECM Screening for in Vivo Repair of Alveolar Bone Defect Using Cell-Laden, Injectable and Photocrosslinkable Hydrogels. ACS Biomater. Sci. Eng..

[72] Klotz B.J., Gawlitta D., Rosenberg A.J., Malda J., Melchels F.P. (2016). Gelatin-Methacryloyl Hydrogels: Towards Biofabrication-Based Tissue Repair. Trends Biotechnol..

[73] Sun M., Sun X., Wang Z., Guo S., Yu G., Yang H. (2018). Synthesis and Properties of Gelatin Methacryloyl (GelMA) Hydrogels and Their Recent Applications in Load-Bearing Tissue. Polymers.

[74] Fahmy S.H., Hassanien E.E.S., Nagy M.M., El Batouty K.M., Mekhemar M., Fawzy El Sayed K., Hassanein E.H., Wiltfang J., Dörfer C. (2017). Investigation of the Regenerative Potential of Necrotic Mature Teeth Following Different Revascularisation Protocols. Aust. Endod. J..

[75] Fawzy El-Sayed K.M., Elsalawy R., Ibrahim N., Gadalla M., Albargasy H., Zahra N., Mokhtar S., El Nahhas N., El Kaliouby Y., Dörfer C.E. (2019). The Dental Pulp Stem/Progenitor Cells-Mediated Inflammatory-Regenerative Axis. Tissue Eng. Part B Rev..

[76] El-Sayed K.M.F., Klingebiel P., Dörfer C.E. (2016). Toll-like Receptor Expression Profile of Human Dental Pulp Stem/Progenitor Cells. J. Endod..

[77] Jakovljevic A., Nikolic N., Jacimovic J., Pavlovic O., Milicic B., Beljic-Ivanovic K., Miletic M., Andric M., Milasin J. (2020). Prevalence of Apical Periodontitis and Conventional Nonsurgical Root Canal Treatment in General Adult Population: An Updated Systematic Review and Meta-Analysis of Cross-Sectional Studies Published between 2012 and 2020. J. Endod..

[78] Lempel E., Lovász B.V., Bihari E., Krajczár K., Jeges S., Tóth Á., Szalma J. (2019). Long-Term Clinical Evaluation of Direct Resin Composite Restorations in Vital vs. Endodontically Treated Posterior Teeth—Retrospective Study up to 13 Years. Dent. Mater..

[79] Murray P.E., Garcia-Godoy F., Hargreaves K.M. (2007). Regenerative Endodontics: A Review of Current Status and a Call for Action. J. Endod..

[80] Sui B., Chen C., Kou X., Li B., Xuan K., Shi S., Jin Y. (2019). Pulp Stem Cell–Mediated Functional Pulp Regeneration. J. Dent. Res..

[81] Cao Y., Song M., Kim E., Shon W., Chugal N., Bogen G., Lin L., Kim R., Park N.H., Kang M. (2015). Pulp-Dentin Regeneration: Current State and Future Prospects. J. Dent. Res..

[82] Huang X., Li Z., Liu A., Liu X., Guo H., Wu M., Yang X., Han B., Xuan K. (2021). Microenvironment Influences Odontogenic Mesenchymal Stem Cells Mediated Dental Pulp Regeneration. Front. Physiol..

[83] Ma P., Chen Y., Lai X., Zheng J., Ye E., Loh X.J., Zhao Y., Parikh B.H., Su X., You M. (2021). The Translational Application of Hydrogel for Organoid Technology: Challenges and Future Perspectives. Macromol. Biosci..

[84] Pankajakshan D., Voytik-Harbin S.L., Nör J.E., Bottino M.C. (2020). Injectable Highly Tunable Oligomeric Collagen Matrices for Dental Tissue Regeneration. ACS Appl. Bio. Mater..

[85] Souron J.-B., Petiet A., Decup F., Tran X.V., Lesieur J., Poliard A., Le Guludec D., Letourneur D., Chaussain C., Rouzet F. (2014). Pulp Cell Tracking by Radionuclide Imaging for Dental Tissue Engineering. Tissue Eng. Part C Methods.

[86] Kim H., Koh W.G., Lee H.G. (2021). Effects of Basic Fibroblast Growth Factor Combined with an Injectable in Situ Crosslinked Hyaluronic Acid Hydrogel for a Dermal Filler. React. Funct. Polym..

[87] Park S.H., Park J.Y., Ji Y.B., Ju H.J., Min B.H., Kim M.S. (2020). An Injectable Click-Crosslinked Hyaluronic Acid Hydrogel Modified with a BMP-2 Mimetic Peptide as a Bone Tissue Engineering Scaffold. Acta Biomater..

[88] Yang R., Tan L., Cen L., Zhang Z. (2016). An Injectable Scaffold Based on Crosslinked Hyaluronic Acid Gel for Tissue Regeneration. RSC Adv..

[89] Silva C.R., Babo P.S., Gulino M., Costa L., Oliveira J.M., Silva-Correia J., Domingues R.M., Reis R.L., Gomes M.E. (2018). Injectable and Tunable Hyaluronic Acid Hydrogels Releasing Chemotactic and Angiogenic Growth Factors for Endodontic Regeneration. Acta Biomater..

[90] El Ashiry E.A., Alamoudi N.M., El Ashiry M.K., Bastawy H.A., El Derwi D.A., Atta H.M. (2018). Tissue Engineering of Necrotic Dental Pulp of Immature Teeth with Apical Periodontitis in Dogs: Radiographic and Histological Evaluation. J. Clin. Pediatr. Dent..

[91] Park S.J., Li Z., Hwang I.N., Huh K.M., Min K.S. (2013). Glycol Chitin–Based Thermoresponsive Hydrogel Scaffold Supplemented with Enamel Matrix Derivative Promotes Odontogenic Differentiation of Human Dental Pulp Cells. J. Endod..

[92] Palma P.J., Ramos J.C., Martins J.B., Diogenes A., Figueiredo M.H., Ferreira P., Viegas C., Santos J.M. (2017). Histologic Evaluation of Regenerative Endodontic Procedures with the Use of Chitosan Scaffolds in Immature Dog Teeth with Apical Periodontitis. J. Endod..

[93] Kim N.R., Lee D.H., Chung P.H., Yang H.C. (2009). Distinct Differentiation Properties of Human Dental Pulp Cells on Collagen, Gelatin, and Chitosan Scaffolds. Oral Surg. Oral Med. Oral Pathol. Oral Radiol. Endodontol..

[94] Hamdy T. (2018). Polymers and Ceramics Biomaterials in Orthopedics and Dentistry: A Review Article. Egypt. J. Chem..

[95] Holiel A.A., Mahmoud E.M., Abdel-Fattah W.M. (2021). Tomographic Evaluation of Direct Pulp Capping Using a Novel Injectable Treated Dentin Matrix Hydrogel: A 2-Year Randomized Controlled Clinical Trial. Clin. Oral Investig..

[96] Kuang R., Zhang Z., Jin X., Hu J., Gupte M.J., Ni L., Ma P.X. (2015). Nanofibrous Spongy Microspheres Enhance Odontogenic Differentiation of Human Dental Pulp Stem Cells. Adv. Healthc. Mater..

[97] Khayat A., Monteiro N., Smith E., Pagni S., Zhang W., Khademhosseini A., Yelick P. (2017). GelMA-Encapsulated HDPSCs and HUVECs for Dental Pulp Regeneration. J. Dent. Res..

[98] Slots J. (2017). Periodontitis: Facts, Fallacies and the Future. Periodontology.

[99] Wang Y., Wang Z., Dong Y. (2023). Collagen-Based Biomaterials for Tissue Engineering. ACS Biomater. Sci. Eng..

[100] Hajishengallis G., Chavakis T. (2021). Local and Systemic Mechanisms Linking Periodontal Disease and Inflammatory Comorbidities. Nat. Rev. Immunol..

[101] Chen M.X., Zhong Y.J., Dong Q.Q., Wong H.M., Wen Y.F. (2021). Global, Regional, and National Burden of Severe Periodontitis, 1990–2019: An Analysis of the Global Burden of Disease Study 2019. J. Clin. Periodontol..

[102] Aljateeli M., Koticha T., Bashutski J., Sugai J.V., Braun T.M., Giannobile W.V., Wang H.L. (2014). Surgical Periodontal Therapy with and without Initial Scaling and Root Planing in the Management of Chronic Periodontitis: A Randomized Clinical Trial. J. Clin. Periodontol..

[103] Liu J., Ruan J., Weir M.D., Ren K., Schneider A., Wang P., Oates T.W., Chang X., Xu H.H. (2019). Periodontal Bone-Ligament-Cementum Regeneration via Scaffolds and Stem Cells. Cells.

[104] Salar Amoli M., EzEldeen M., Jacobs R., Bloemen V. (2022). Materials for Dentoalveolar Bioprinting: Current State of the Art. Biomedicines.

[105] Chen F.M., Jin Y. (2010). Periodontal Tissue Engineering and Regeneration: Current Approaches and Expanding Opportunities. Tissue Eng. Part B Rev..

[106] Ma P., Wang Z., Jiang Y., Huang Z., Xia L., Jiang J., Yuan F., Xia H., Zhang Y. (2022). Clay-Based Nanocomposite Hydrogels with Microstructures and Sustained Ozone Release for Antibacterial Activity. Colloids Surf. A Physicochem. Eng. Asp..

[107] Butera A., Pascadopoli M., Gallo S., Pérez-Albacete Martínez C., Maté Sánchez de Val J.E., Parisi L., Gariboldi A., Scribante A. (2023). Ozonized Hydrogels vs. 1% Chlorhexidine Gel for the Clinical and Domiciliary Management of Peri-Implant Mucositis: A Randomized Clinical Trial. J. Clin. Med..

[108] Cannizzaro S., Maiorani C., Scribante A., Butera A. (2023). Personalized Treatment of Periodontitis in a Patient with McArdle’s Disease: The Benefits from Probiotics. Case Rep. Dent..

[109] Butera A., Pascadopoli M., Gallo S., Alovisi M., Lovati E., Mutti E., Scribante A. (2022). Domiciliary Management of Periodontal Indexes and Glycosylated Hemoglobin (HbA1c) in Type 1 Diabetic Patients with Paraprobiotic-Based Toothpaste and Mousse: Randomized Clinical Trial. Appl. Sci..

[110] Chang B., Ahuja N., Ma C., Liu X. (2017). Injectable Scaffolds: Preparation and Application in Dental and Craniofacial Regeneration. Mater. Sci. Eng. R Rep..

[111] Fan C., Wang D.A. (2017). Macroporous Hydrogel Scaffolds for Three-Dimensional Cell Culture and Tissue Engineering. Tissue Eng. Part B Rev..

[112] Mantha S., Pillai S., Khayambashi P., Upadhyay A., Zhang Y., Tao O., Pham H.M., Tran S.D. (2019). Smart Hydrogels in Tissue Engineering and Regenerative Medicine. Materials.

[113] Yang J.M., Olanrele O.S., Zhang X., Hsu C.C. (2018). Fabrication of Hydrogel Materials for Biomedical Applications. Nov. Biomater. Regen. Med..

[114] Jung I.H., Park J.C., Kim J.C., Jeon D.W., Choi S.H., Cho K.S., Im G.I., Kim B.S., Kim C.S. (2012). Novel Application of Human Periodontal Ligament Stem Cells and Water-Soluble Chitin for Collagen Tissue Regeneration: In Vitro and In Vivo Investigations. Tissue Eng. Part A.

[115] Momose T., Miyaji H., Kato A., Ogawa K., Yoshida T., Nishida E., Murakami S., Kosen Y., Sugaya T., Kawanami M. (2016). Collagen Hydrogel Scaffold and Fibroblast Growth Factor-2 Accelerate Periodontal Healing of Class II Furcation Defects in Dog. Open Dent. J..

[116] Burdick J.A., Prestwich G.D. (2011). Hyaluronic Acid Hydrogels for Biomedical Applications. Adv. Mater..

[117] Fraser J.R.E., Laurent T.C., Laurent U. (1997). Hyaluronan: Its Nature, Distribution, Functions and Turnover. J. Intern. Med..

[118] Oksala O., Salo T., Tammi R., Häkkinen L., Jalkanen M., Inki P., Larjava H. (1995). Expression of Proteoglycans and Hyaluronan during Wound Healing. J. Histochem. Cytochem..

[119] Rosaming P., Jirayupapong J., Thamnium S., Win Y.Y., Limprasutr V., Rodsiri R., Pavasant P., Luckanagul J.A. (2022). Interpenetrating Low-Molecular Weight Hyaluronic Acid in Hyaluronic Acid-Based In Situ Hydrogel Scaffold for Periodontal and Oral Wound Applications. Polymers.

[120] Babo P.S., Pires R.L., Santos L., Franco A., Rodrigues F., Leonor I., Reis R.L., Gomes M.E. (2017). Platelet Lysate-Loaded Photocrosslinkable Hyaluronic Acid Hydrogels for Periodontal Endogenous Regenerative Technology. ACS Biomater. Sci. Eng..

[121] Miranda D.G., Malmonge S.M., Campos D.M., Attik N.G., Grosgogeat B., Gritsch K. (2016). A Chitosan-Hyaluronic Acid Hydrogel Scaffold for Periodontal Tissue Engineering. J. Biomed. Mater. Res. B Appl. Biomater..

[122] de Santana R.B., de Santana C.M.M. (2015). Human Intrabony Defect Regeneration with RhFGF-2 and Hyaluronic Acid—A Randomized Controlled Clinical Trial. J. Clin. Periodontol..

[123] Xu X., Gu Z., Chen X., Shi C., Liu C., Liu M., Wang L., Sun M., Zhang K., Liu Q. (2019). An Injectable and Thermosensitive Hydrogel: Promoting Periodontal Regeneration by Controlled-Release of Aspirin and Erythropoietin. Acta Biomater..

[124] Chen F.M., Zhao Y.M., Zhang R., Jin T., Sun H.H., Wu Z.F., Jin Y. (2007). Periodontal Regeneration Using Novel Glycidyl Methacrylated Dextran (Dex-GMA)/Gelatin Scaffolds Containing Microspheres Loaded with Bone Morphogenetic Proteins. J. Control. Release.

[125] Chen L., Shen R., Komasa S., Xue Y., Jin B., Hou Y., Okazaki J., Gao J. (2017). Drug-Loadable Calcium Alginate Hydrogel System for Use in Oral Bone Tissue Repair. Int. J. Mol. Sci..

[126] Zhang Y., Dou X., Zhang L., Wang H., Zhang T., Bai R., Sun Q., Wang X., Yu T., Wu D. (2022). Facile Fabrication of a Biocompatible Composite Gel with Sustained Release of Aspirin for Bone Regeneration. Bioact. Mater..

[127] Zhan H., Löwik D.W. (2019). A Hybrid Peptide Amphiphile Fiber PEG Hydrogel Matrix for 3D Cell Culture. Adv. Funct. Mater..

[128] Fraser D., Benoit D. (2022). Dual Peptide-Functionalized Hydrogels Differentially Control Periodontal Cell Function and Promote Tissue Regeneration. Biomater. Adv..

[129] Pan J., Deng J., Yu L., Wang Y., Zhang W., Han X., Camargo P.H., Wang J., Liu Y. (2020). Investigating the Repair of Alveolar Bone Defects by Gelatin Methacrylate Hydrogels-Encapsulated Human Periodontal Ligament Stem Cells. J. Mater. Sci. Mater. Med..

[130] Kakarala K., Shnayder Y., Tsue T.T., Girod D.A. (2018). Mandibular Reconstruction. Oral Oncol..

[131] Zhang Q., Wu W., Qian C., Xiao W., Zhu H., Guo J., Meng Z., Zhu J., Ge Z., Cui W. (2019). Advanced Biomaterials for Repairing and Reconstruction of Mandibular Defects. Mater. Sci. Eng. C.

[132] Berg B.I., Juergens P., Soerensen Y., Savic M., Zeilhofer H.F., Schwenzer-Zimmerer K. (2014). Traumatology of the Facial Skeleton in Octogenarian Patients: A Retrospective Analysis of 96 Cases. J. Cranio-Maxillofac. Surg..

[133] Thariat J., Julieron M., Brouchet A., Italiano A., Schouman T., Marcy P.Y., Odin G., Lacout A., Dassonville O., Peyrottes-Birstwisles I. (2012). Osteosarcomas of the Mandible: Are They Different from Other Tumor Sites?. Crit. Rev. Oncol. Hematol..

[134] Bede S.Y.H., Ismael W.K., Hashim E.A. (2019). Reconstruction Plate-Related Complications in Mandibular Continuity Defects. Oral Maxillofac. Surg..

[135] Diniz-Freitas M., Fernández-Feijoo J., Diz Dios P., Pousa X., Limeres J. (2018). Denosumab-Related Osteonecrosis of the Jaw Following Non-Surgical Periodontal Therapy: A Case Report. J. Clin. Periodontol..

[136] Joo Y.H., Cho J.K., Koo B.S., Kwon M., Kwon S.K., Kwon S.Y., Kim M.S., Kim J.K., Kim H., Nam I. (2019). Guidelines for the Surgical Management of Oral Cancer: Korean Society of Thyroid-Head and Neck Surgery. Clin. Exp. Otorhinolaryngol..

[137] Armiento A.R., Hatt L.P., Sanchez Rosenberg G., Thompson K., Stoddart M.J. (2020). Functional Biomaterials for Bone Regeneration: A Lesson in Complex Biology. Adv. Funct. Mater..

[138] Guo J., Yao H., Li X., Chang L., Wang Z., Zhu W., Su Y., Qin L., Xu J. (2023). Advanced Hydrogel Systems for Mandibular Reconstruction. Bioact. Mater..

[139] Ning H., Wu X., Wu Q., Yu W., Wang H., Zheng S., Chen Y., Li Y., Su J. (2019). Microfiber-Reinforced Composite Hydrogels Loaded with Rat Adipose-Derived Stem Cells and BMP-2 for the Treatment of Medication-Related Osteonecrosis of the Jaw in a Rat Model. ACS Biomater. Sci. Eng..

[140] Batstone M. (2018). Reconstruction of Major Defects of the Jaws. Aust. Dent. J..

[141] Paré A., Bossard A., Laure B., Weiss P., Gauthier O., Corre P. (2019). Reconstruction of Segmental Mandibular Defects: Current Procedures and Perspectives. Laryngoscope Investig. Otolaryngol..

[142] Al Maruf D.A., Ghosh Y.A., Xin H., Cheng K., Mukherjee P., Crook J.M., Wallace G.G., Klein T.J., Clark J.R. (2022). Hydrogel: A Potential Material for Bone Tissue Engineering Repairing the Segmental Mandibular Defect. Polymers.

[143] Tatara A., Wong M., Mikos A. (2014). In Vivo Bioreactors for Mandibular Reconstruction. J. Dent. Res..

[144] Buwalda S.J., Vermonden T., Hennink W.E. (2017). Hydrogels for Therapeutic Delivery: Current Developments and Future Directions. Biomacromolecules.

[145] Yue S., He H., Li B., Hou T. (2020). Hydrogel as a Biomaterial for Bone Tissue Engineering: A Review. Nanomaterials.

[146] Naahidi S., Jafari M., Logan M., Wang Y., Yuan Y., Bae H., Dixon B., Chen P. (2017). Biocompatibility of Hydrogel-Based Scaffolds for Tissue Engineering Applications. Biotechnol. Adv..

[147] Sun M., Cheng L., Xu Z., Chen L., Liu Y., Xu Y., Zhou D., Zhang X., Zhou Q., Sun J. (2022). Preparation and Characterization of Vancomycin Hydrochloride-Loaded Mesoporous Silica Composite Hydrogels. Front. Bioeng. Biotechnol..

[148] Thabit A.K., Fatani D.F., Bamakhrama M.S., Barnawi O.A., Basudan L.O., Alhejaili S.F. (2019). Antibiotic Penetration into Bone and Joints: An Updated Review. Int. J. Infect. Dis..

[149] Sungkhaphan P., Thavornyutikarn B., Kaewkong P., Pongkittiphan V., Pornsuwan S., Singhatanadgit W., Janvikul W. (2021). Antibacterial and Osteogenic Activities of Clindamycin-Releasing Mesoporous Silica/Carboxymethyl Chitosan Composite Hydrogels. R. Soc. Open Sci..

[150] Hamdy T.M. (2023). Dental Biomaterial Scaffolds in Tooth Tissue Engineering: A Review. Curr. Oral Health Rep..

[151] Zhang Y., Liu Z., Chen A., Wang Q., Zhang J., Zhao C., Xu J., Yang W., Peng Y., Zhang Z. (2020). Fabrication of Micro-/Submicro-/Nanostructured Polypropylene/Graphene Superhydrophobic Surfaces with Extreme Dynamic Pressure Resistance Assisted by Single Hierarchically Porous Anodic Aluminum Oxide Template. J. Phys. Chem. C.

[152] Wang Q., Chen A., Gu H., Qin G., Zhang J., Xu J., Jiang G., Liu W., Zhang Z., Huang H. (2021). Highly Interconnected Porous PDMS/CNTs Sandwich Sponges with Anti-Icing/Deicing Microstructured Surfaces. J. Mater. Sci..

[153] Chen A., Wang Q., Li M., Peng Z., Lai J., Zhang J., Xu J., Huang H., Lei C. (2021). Combined Approach of Compression Molding and Magnetic Attraction to Micropatterning of Magnetic Polydimethylsiloxane Composite Surfaces with Excellent Anti-Icing/Deicing Performance. ACS Appl. Mater. Interfaces.

[154] Kumar P.S., Hashimi S., Saifzadeh S., Ivanovski S., Vaquette C. (2018). Additively Manufactured Biphasic Construct Loaded with BMP-2 for Vertical Bone Regeneration: A Pilot Study in Rabbit. Mater. Sci. Eng. C.

[155] Vaquette C., Mitchell J., Fernandez-Medina T., Kumar S., Ivanovski S. (2021). Resorbable Additively Manufactured Scaffold Imparts Dimensional Stability to Extraskeletally Regenerated Bone. Biomaterials.

[156] Suo L., Xue Z., Wang P., Wu H., Chen Y., Shen J. (2022). Improvement of Osteogenic Properties Using a 3D-Printed Graphene Oxide/Hyaluronic Acid/Chitosan Composite Scaffold. J. Bioact. Compat. Polym..

[157] Lei L., Liu Z., Yuan P., Jin R., Wang X., Jiang T., Chen X. (2019). Injectable Colloidal Hydrogel with Mesoporous Silica Nanoparticles for Sustained Co-Release of MicroRNA-222 and Aspirin to Achieve Innervated Bone Regeneration in Rat Mandibular Defects. J. Mater. Chem. B.

[158] Mehrabani D., Khodakaram-Tafti A., Shaterzadeh-Yazdi H., Zamiri B., Omidi M. (2018). Comparison of the Regenerative Effect of Adipose-Derived Stem Cells, Fibrin Glue Scaffold, and Autologous Bone Graft in Experimental Mandibular Defect in Rabbit. Dent. Traumatol..

[159] Liu T., Xu J., Pan X., Ding Z., Xie H., Wang X., Xie H. (2021). Advances of Adipose-Derived Mesenchymal Stem Cells-Based Biomaterial Scaffolds for Oral and Maxillofacial Tissue Engineering. Bioact. Mater..

[160] Jung R.E., Hälg G.A., Thoma D.S., Hämmerle C.H.F. (2009). A Randomized, Controlled Clinical Trial to Evaluate a New Membrane for Guided Bone Regeneration around Dental Implants. Clin. Oral Implant. Res..

[161] Trubelja A., Kasper F.K., Farach-Carson M.C., Harrington D.A. (2022). Bringing Hydrogel-Based Craniofacial Therapies to the Clinic. Acta Biomater..

[162] Ali Salim K.M., Abd Jalil A., Radzi Z., Ismail S.M., Czernuszka J.T., Rahman M.T. (2020). Inflammatory Responses in Oro-Maxillofacial Region Expanded Using Anisotropic Hydrogel Tissue Expander. Materials.

[163] Su T., Zheng A., Cao L., Peng L., Wang X., Wang J., Xin X., Jiang X. (2022). Adhesion-Enhancing Coating Embedded with Osteogenesis-Promoting PDA/HA Nanoparticles for Peri-Implant Soft Tissue Sealing and Osseointegration. Bio-Des. Manuf..

[164] Han G., Ceilley R. (2017). Chronic Wound Healing: A Review of Current Management and Treatments. Adv. Ther..

[165] Ajovalasit A., Redondo-Gomez C., Sabatino M.A., Okesola B.O., Braun K., Mata A., Dispenza C. (2021). Carboxylated-Xyloglucan and Peptide Amphiphile Co-Assembly in Wound Healing. Regen. Biomater..

[166] Peng L., Chang L., Si M., Lin J., Wei Y., Wang S., Liu H., Han B., Jiang L. (2020). Hydrogel-Coated Dental Device with Adhesion-Inhibiting and Colony-Suppressing Properties. ACS Appl. Mater. Interfaces.

[167] Pei B., Wang W., Fan Y., Wang X., Watari F., Li X. (2017). Fiber-Reinforced Scaffolds in Soft Tissue Engineering. Regen. Biomater..

[168] Kuth S., Karakaya E., Reiter N., Schmidt L., Paulsen F., Teßmar J., Budday S., Boccaccini A.R. (2022). Oxidized Hyaluronic Acid-Gelatin-Based Hydrogels for Tissue Engineering and Soft Tissue Mimicking. Tissue Eng. Part C Methods.

[169] Wu J., Pan Z., Zhao Z.Y., Wang M.H., Dong L., Gao H.L., Liu C.Y., Zhou P., Chen L., Shi C.J. (2022). Anti-Swelling, Robust, and Adhesive Extracellular Matrix-Mimicking Hydrogel Used as Intraoral Dressing. Adv. Mater..

[170] Cui W., King D.R., Huang Y., Chen L., Sun T.L., Guo Y., Saruwatari Y., Hui C.Y., Kurokawa T., Gong J.P. (2020). Fiber-Reinforced Viscoelastomers Show Extraordinary Crack Resistance That Exceeds Metals. Adv. Mater..

[171] Stubbe B., Mignon A., Declercq H., Van Vlierberghe S., Dubruel P. (2019). Development of Gelatin-Alginate Hydrogels for Burn Wound Treatment. Macromol. Biosci..

[172] Guo Y., He M., Peng Y., Zhang Q., Yan L., Zan X. (2020). κ-Carrageenan/Poly(N-Acryloyl Glycinamide) Double-Network Hydrogels with High Strength, Good Self-Recovery, and Low Cytotoxicity. J. Mater. Sci..

[173] Erdagi S.I., Ngwabebhoh F.A., Yildiz U. (2020). Genipin Crosslinked Gelatin-Diosgenin-Nanocellulose Hydrogels for Potential Wound Dressing and Healing Applications. Int. J. Biol. Macromol..

[174] Edmans J.G., Clitherow K.H., Murdoch C., Hatton P.V., Spain S.G., Colley H.E. (2020). Mucoadhesive Electrospun Fibre-Based Technologies for Oral Medicine. Pharmaceutics.

[175] Fonseca-Santos B., Chorilli M. (2018). An Overview of Polymeric Dosage Forms in Buccal Drug Delivery: State of Art, Design of Formulations and Their in Vivo Performance Evaluation. Mater. Sci. Eng. C.

[176] Liang Y., Li Z., Huang Y., Yu R., Guo B. (2021). Dual-Dynamic-Bond Cross-Linked Antibacterial Adhesive Hydrogel Sealants with on-Demand Removability for Post-Wound-Closure and Infected Wound Healing. ACS Nano.

[177] Zheng W., Hao Y., Wang D., Huang H., Guo F., Sun Z., Shen P., Sui K., Yuan C., Zhou Q. (2021). Preparation of Triamcinolone Acetonide-Loaded Chitosan/Fucoidan Hydrogel and Its Potential Application as an Oral Mucosa Patch. Carbohydr. Polym..

[178] Ansari S., Pouraghaei Sevari S., Chen C., Sarrion P., Moshaverinia A. (2021). RGD-Modified Alginate–GelMA Hydrogel Sheet Containing Gingival Mesenchymal Stem Cells: A Unique Platform for Wound Healing and Soft Tissue Regeneration. ACS Biomater. Sci. Eng..

[179] Yi K., Li Q., Lian X., Wang Y., Tang Z. (2022). Utilizing 3D Bioprinted Platelet-Rich Fibrin-Based Materials to Promote the Regeneration of Oral Soft Tissue. Regen. Biomater..

[180] Zhang W., Bao B., Jiang F., Zhang Y., Zhou R., Lu Y., Lin S., Lin Q., Jiang X., Zhu L. (2021). Promoting Oral Mucosal Wound Healing with a Hydrogel Adhesive Based on a Phototriggered S-Nitrosylation Coupling Reaction. Adv. Mater..

[181] Qi W., Dong N., Wu L., Zhang X., Li H., Wu H., Ward N., Yu J., Liu H., Wang J. (2023). Promoting Oral Mucosal Wound Healing Using a DCS-RuB2A2 Hydrogel Based on a Photoreactive Antibacterial and Sustained Release of BMSCs. Bioact. Mater..

[182] Kim S.Y., Hwang Y.-S., Chun H.J., Yang D.H. (2020). Preparation of a Photocured GelMA Hydrogel Co-Cultured with HOKs and HGFs for an Artificial Oral Mucosal Tissue Model. J. Ind. Eng. Chem..

[183] Wang X., Yuan Z., Tao A., Wang P., Xie W., Yang S., Huang J., Wen N. (2022). Hydrogel-Based Patient-Friendly Photodynamic Therapy of Oral Potentially Malignant Disorders. Biomaterials.

[184] Ryu J.H., Choi J.S., Park E., Eom M.R., Jo S., Lee M.S., Kwon S.K., Lee H. (2020). Chitosan Oral Patches Inspired by Mussel Adhesion. J. Control. Release.

[185] ClinicalTrials.gov. https://clinicaltrials.gov/ct2/results?cond=oral&term=hydrogel&cntry=&state=&city=&dist=.

